# N-terminal thrombospondin-1 enhances muscle function and tissue-specific metabolic adaptation in response to exercise and cold exposure

**DOI:** 10.1038/s12276-026-01830-z

**Published:** 2026-09-08

**Authors:** Min Ju Kim, Hyun-Jun Jang, Jae Sook Kang, Sung-Wook Chun, Sun Gun Chung, Shinyeong Ju, ByungKuk Min, Eunhwan Kim, Ju Yeon Kwak, Ji-Won Kim, Seongwan Kim, Yeo Jin Shin, Kwang-Pyo Lee, Cheolju Lee, Seung-Min Lee, Jung Jae Won, Eun-Soo Kwon, Jang Hyun Choi, Ki-Sun Kwon, Yong Ryoul Yang

**Affiliations:** 1 Aging Convergence Research Center, Korea Research Institute of Bioscience and Biotechnology (KRIBB), Daejeon, 34141 Republic of Korea; 2 Department of Bio-molecular Science, KRIBB School of Bioscience, Korea University of Science and Technology (UST), Daejeon, 34113 Republic of Korea; 3 Research Group of Personalized Diet, Korea Food Research Institute, Wanju-gun, Jeollabuk-do 55365 Republic of Korea; 4 Department of Biological Sciences, Ulsan National Institute of Science and Technology (UNIST), Ulsan, 44919 Republic of Korea; 5 Research Institute of Physical Education & Sports Science, Pusan National University, Pusan, Republic of Korea; 6 Department of Rehabilitation Medicine, Seoul National University College of Medicine, Seoul, Republic of Korea; 7 Institute of Aging, Seoul National University, Seoul, Republic of Korea; 8 Chemical and Biological Integrative Research Center, Korea Institute of Science and Technology, Seoul, 02792 Republic of Korea; 9 Stem Cell Research Center, Korea Research Institute of Bioscience and Biotechnology (KRIBB), Daejeon, 34141 Republic of Korea; 10 Aventi Inc., Daejeon, 34141 Republic of Korea; 11 Division of Bio-Medical Science & Technology, KIST School, University of Science and Technology, Seoul, 02792 Republic of Korea; 12 Metadium Therapeutics, Ulsan, 44919 Republic of Korea

**Keywords:** Metabolism, Extracellular signalling molecules, Cell signalling

## Abstract

Exercise and cold exposure elicit partly overlapping metabolic adaptations through distinct mechanisms. Here, we sought to identify circulating factors shared by these stimuli. We found that exercise and cold exposure upregulated thrombospondin-1 (TSP1) expression in skeletal muscle and brown adipose tissue (BAT) and induced the release of its N-terminal fragment (N-TSP1). In high-fat-diet-induced obese mice, N-TSP1 administration enhanced muscle strength, increased energy expenditure and attenuated insulin resistance and hepatic steatosis. N-TSP1 also increased mitochondrial respiration and oxidative metabolism in skeletal muscle and stimulated thermogenic marker gene expression in brown adipocytes. In aged mice, N-TSP1 administration significantly improved physical performance and was associated with tissue-specific metabolic remodelling. In humans, plasma N-TSP1 levels correlated positively with muscle mass and physical performance. Together, these findings support a role for N-TSP1 as an exercise- and cold-responsive circulating factor that modulates skeletal muscle function and tissue-specific metabolic adaptation, with potential relevance to obesity- and age-related functional decline.

**Figure:**
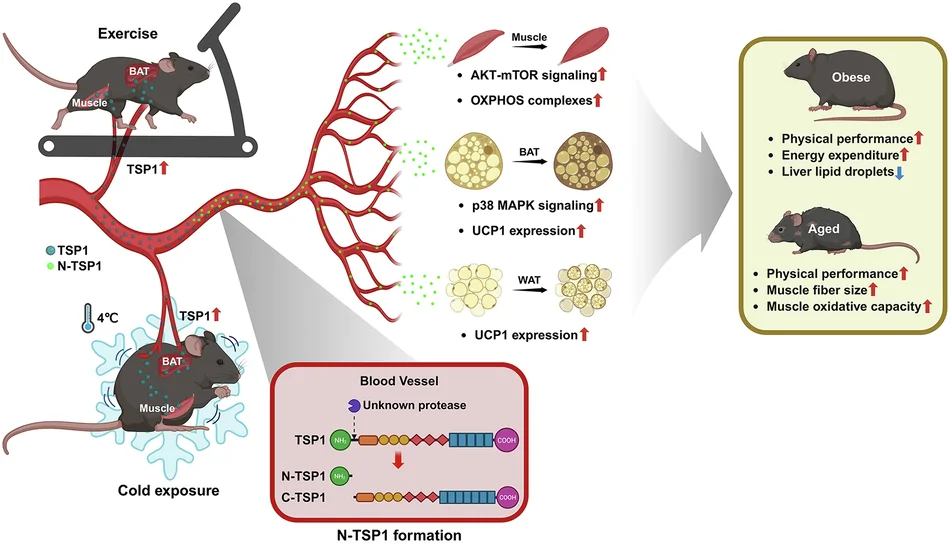

## N-TSP1 Links Exercise and Cold Exposure to Metabolic Health

Exercise and cold exposure enhance metabolic health, yet the underlying mechanisms remain incompletely understood. Using transcriptome and proteome analyses, this study identifies thrombospondin-1 (TSP1) as a factor commonly induced by both stimuli in humans and rodents. Exercise and cold exposure increased TSP1 levels in skeletal muscle and brown adipose tissue, with the N-terminal fragment (N-TSP1) emerging as a key mediator. N-TSP1 enhanced thermogenesis in adipocytes and improved mitochondrial function in muscle cells. In mice fed a high-fat diet, N-TSP1 improved glucose tolerance and insulin sensitivity. In aged mice, N-TSP1 enhanced muscle strength and endurance, accompanied by molecular changes consistent with tissue-specific metabolic adaptation. Together, these findings establish N-TSP1 as a mediator of the metabolic benefits induced by exercise and cold exposure and highlight its potential in metabolic disease management.

This summary was initially drafted using artificial intelligence, then revised and fact-checked by the author.

## Introduction

Exercise is a key non-pharmacological intervention for healthy ageing, as it strengthens bones, reduces risks of diseases such as heart disease, depression, certain cancers and dementia, and improves healthspan. Cold exposure also offers potential benefits for circulation, metabolism^1^ and immune function^2,3^, with moderate reductions in core body temperature linked to lifespan extension in worms, rodents^4,5^ and humans^6^. Cold exposure primarily activates brown adipose tissue (BAT) through mitochondrial uncoupling, which boosts glucose and lipid uptake to improve energy expenditure and insulin sensitivity^7^. By contrast, exercise broadly enhances systemic metabolism by reducing adipocyte size and lipid content, increasing mitochondrial activity, and inducing browning of white adipose tissue (WAT)^8^. Of note, both exercise and cold exposure induce the release of specific circulating factors within the body. 12,13-diHOME is released from BAT following cold exposure in both rodents (at 4°C) and humans (at 14°C)^9^. In addition, acute exercise elevates circulating 12,13-diHOME in humans and mice^10^. This release of 12,13-diHOME enhances skeletal muscle fatty acid uptake and oxidation, as well as BAT thermogenic activity. Irisin, a myokine induced by exercise, facilitates energy expenditure by upregulating uncoupling protein 1 (UCP1) levels, thereby inducing WAT browning^11^. Notably, Lee et al. demonstrated that irisin is also released in humans upon stimulation by shivering, suggesting a possible evolutionary link between exercise-induced irisin secretion and shivering-related muscle contraction^12^. We hypothesized that both exercise and cold exposure, which trigger skeletal muscle contraction, promote the release of circulating factors with shared metabolic functions. By integrating published transcriptome data from human skeletal muscle and plasma proteomics from exercise-trained rodents, we identified thrombospondin-1 (TSP1) as a commonly upregulated gene. The thrombospondin (TSP) family comprises five members (TSP1, TSP2, TSP3, TSP4 and TSP5) and each member of the family exhibits distinct expression patterns, tissue localization and functions^13^. TSP1 is the most well-studied member, known for its roles in blood clotting, wound healing and angiogenesis^14^. Although its trimeric structure is crucial for function, proteolytic cleavage produces fragments with unique roles. For example, the N-terminal fragment of TSP1 (N-TSP1) generated by HtrA1 promotes the formation of tube-like structures by endothelial cells^15^. Cleavage of TSP1 by BMP-1 significantly increases its capacity to induce differentiation of keratocytes into myofibroblasts during corneal wound healing^16^.

Our study reveal**s** a novel role for N-TSP1 as a circulating factor induced by both exercise and cold exposure. Although N-TSP1 shares induction patterns with exercise- and cold-responsive factors such as irisin, its functional role appears to be that of a context-specific modulator of metabolic adaptation rather than a universal mimetic of cold-induced thermogenesis. Using in vitro and complementary in vivo models, we provide evidence that circulating N-TSP1 supports skeletal muscle function and tissue-specific metabolic remodelling associated with exercise and cold exposure.

## Materials and methods

### Animals and diets

C57BL/6J male mice were used for all experiments. Young mice (12–16 weeks old) and aged mice (20–23 months old) were obtained from the Laboratory Animal Resource Centre (LARC) at KRIBB. Animals were housed in individually ventilated cages maintained at 22–24°C under a 12-h light/dark cycle. Standard rodent chow and water were provided ad libitum. Mice were fed either normal chow (% kcal: protein 20.5, carbohydrate 69.1, fat 10.5; TD.08806 Envigo) or a high-fat diet (HFD; % kcal: protein 18.3, carbohydrate 21.4, fat 60.3; TD.06414, Envigo).

### Recombinant TSP1 proteins

Protein production was carried out by Y-Biologics (Daejeon, South Korea). Briefly, sequences encoding mouse N-TSP1 (aa 19–259), middle fragment of TSP1 (M-TSP1; aa 260–690) and C-terminal fragment of TSP1 (C-TSP1; aa 691–1170) were ordered as a synthesized product (GeneArt, Thermo Fisher Scientific) and cloned into a Y-Biologics expression vector containing an N-terminal IgG Fc tag, such that each fragment was expressed as an Fc-fusion recombinant protein. Each recombinant protein was targeted for secretion by the addition of the signal peptide sequence MGWSYIILFLVATAADVHS at its N-terminus. FreeStyle 293-F Cells (R79007, Thermo) were used to produce recombinant proteins. Each DNA construct was transfected into HEK293F cells and cultured in a shaking incubator at 37°C, 8% CO_2_, and 125 rpm for 24 h. After 24 h of culture, 10% Soytone was added to the culture medium to a final concentration of 0.5%, and the cells were further incubated at 37°C, 8% CO_2_, and 125 rpm for an additional 120 h. Six days post-transfection, the HEK293F cells were harvested by centrifugation at 3,800 rpm for 30 min. The supernatant was collected for protein purification, and a primary purification step was performed using protein A affinity chromatography. The purity and molecular weight of the purified protein were analysed by SDS-PAGE and size-exclusion chromatography–high-performance liquid chromatography (SEC-HPLC). Human recombinant N-TSP1 protein (Ser24–Thr221) (RPA611Hu01, USCN) and recombinant mouse full-length TSP1 protein (F-TSP1; 7859-TH, R&D Systems) were purchased from a commercial protein supplier. PBS was used as the vehicle control for all in vitro and in vivo treatments.

### Skeletal muscle function test

#### Time and distance to exhaustion assessment

Following a 5-day habituation period, mice were subjected to a running capacity test to assess their time to exhaustion. The treadmill protocol began with a low-intensity phase (5 m/min for 5 min), followed by a progressive increase in speed (2 m/min every 2 min) on a 10% incline. Exhaustion was defined as exceeding 10 s of continuous contact with the shock grid.

#### Strength evaluation in the grip test

Forelimb and hindlimb grip strength were assessed using an angled grid. Mice were pulled backward in triplicate trials, and the peak force (Newtons) exerted before losing grip was recorded. The average force was then normalized to individual body weight (BW).

#### Inverted grid test

Mice were placed on a centralized platform within a wire mesh apparatus. The apparatus was then flipped, suspending the mice 40 cm above a cushioned surface. Grip strength was evaluated by recording the latency to fall (time spent clinging).

### Human cohorts

#### Young adult cohort

Study participants comprised ten young, active male volunteers with the following characteristics (mean ± SD): age 24.13 ± 1.457 years, height 177.3 ± 1.812 cm and body mass 74.28 ± 3.181 kg. The exercise interventions included: (1) moderate-intensity resistance training at 50–69% of individual one-repetition maximum (1RM), and (2) moderate-intensity endurance exercise consisting of treadmill running for 60 min at 40–59% of maximum heart rate (HRmax). Participants maintained an 8-h fasting state prior to each exercise session. Blood samples were collected at two time points: immediately before exercise and 1 h after exercise completion. The study was approved by the Institutional Review Board (IRB) at Pusan National University (IRB protocol number PNU_2021_140_HR).

#### Elderly cohort

Plasma samples were collected from randomly selected older female participants (*n* = 14; mean age, 75.0 ± 3.46 years, mean ± SD). The study was approved by the Institutional Review Board (IRB) at Seoul National University (IRB protocol number 17-2018-0024). All participants provided written informed consent.

## Results

### Exercise and cold exposure upregulate thrombospondin-1

**Fig. 1:**
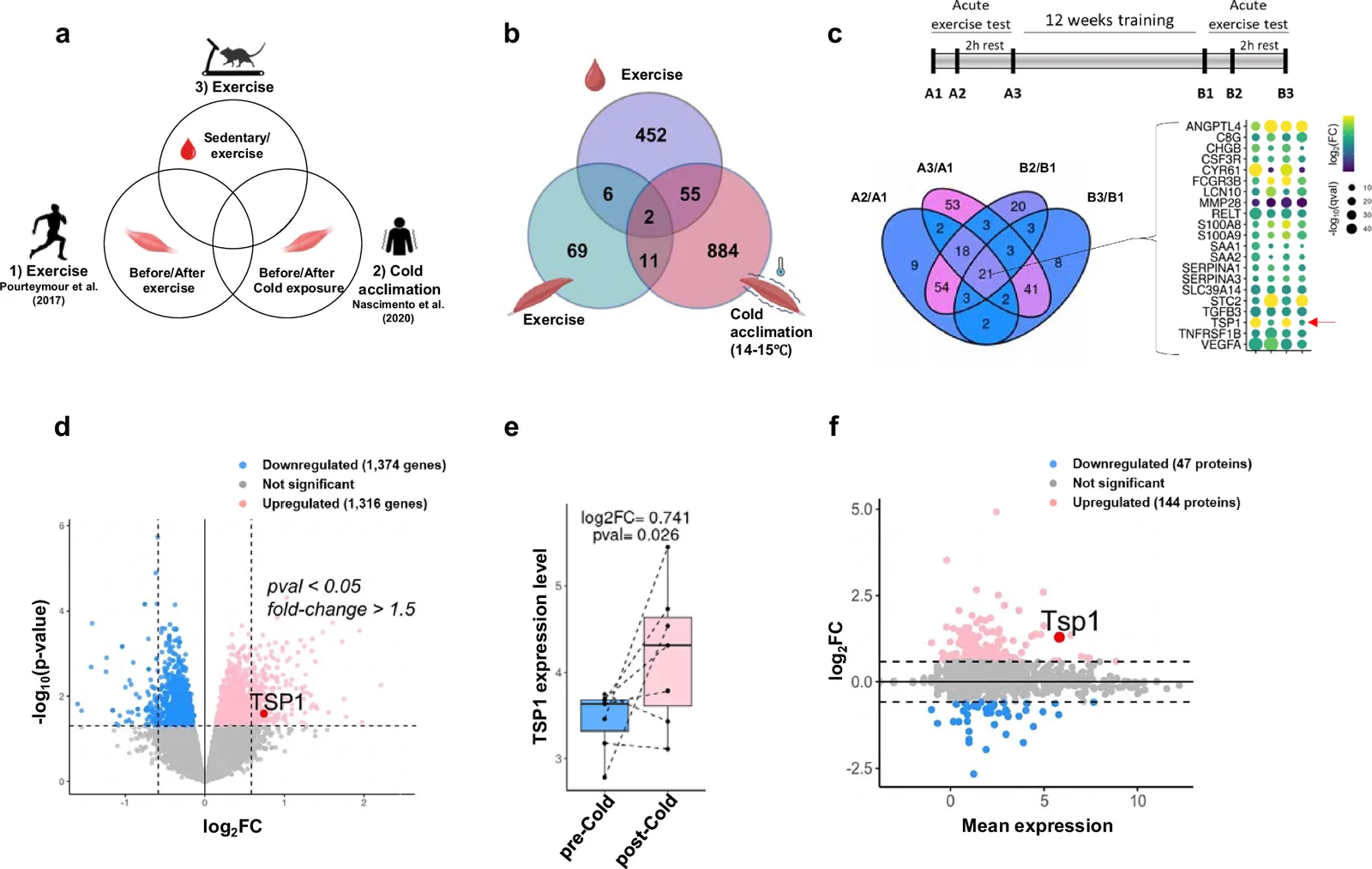
TSP1 is upregulated in skeletal muscle and plasma after exercise and cold exposure. **a** Schematic overview of the workflow used to identify common secreted factors across the three datasets. **b** Venn diagram of overlapping differentially expressed genes (DEGs) across datasets, visualizing upregulated genes. **c** Exercise programme design with biopsy time points (A1–3, B1–3) (upper). Venn diagrams of upregulated secretory genes (lower). Dot plot displays the log_2_(fold change, FC) values and q-values for common DEGs. Dot colours represent the log_2_FC; dot size indicates the −log_10_(q-value). The red arrow highlights TSP1. **d** Volcano plot of before (Pre) and after (Post) cold exposure DEGs. Blue = downregulated, grey = unchanged, red = upregulated. Vertical dashed lines: log_2_FC cutoff (± 1); horizontal dashed line: statistical significance threshold (*P* = 0.05). **e** Expression levels of TSP1 in human skeletal muscle before (Pre) and after (post) cold exposure. Dashed lines connect individual samples (*n* = 8 per group). **f** MA plot of before (Pre) and after (Post) exercise differentially secreted proteins in mice. Blue = downregulated, grey = unchanged, red = upregulated. Dashed lines: log_2_FC cutoff (± 1).

To identify circulating factors secreted from contracted muscle cells in response to exercise and cold exposure, we performed a comparative multi-omics analysis. This analysis integrated datasets from three independent models (Fig. 1a): 1) the transcriptome profiling of human vastus lateralis muscle following acute exercise before and after 12 weeks of cycle training^17^ (Supplementary Dataset 1), 2) changes in the transcriptome of human vastus lateralis muscle after 10 days of cold acclimation (14–15°C) for 2–6 h daily^18^ (Supplementary Dataset 2, GSE156249) and 3) plasma proteome profiling of exercised mice (Supplementary Dataset 3). We focused on transcripts that encode secretory proteins upregulated in all datasets. Integrative analysis of three datasets identified TSP1 and tenascin-C (TNC) as candidate factors consistently upregulated in all three models (Fig. 1b). Interestingly, TSP1 and TNC are both extracellular matrix glycoproteins that play important roles in various biological processes, including tissue remodelling, cell adhesion and migration^19,20^. Despite these similarities, TNC and TSP1 also exhibit distinct functions and roles in specific tissues and contexts. TNC plays a crucial role in muscle repair following mechanical damage. Recent studies have further demonstrated that TNC serum levels increase in response to anaerobic exercise, suggesting a potential link between exercise and enhanced repair processes^21,22^. However, whether TNC is regulated by cold exposure remains unclear. In contrast to the relatively established role of TNC in muscle repair, the role of TSP1 in exercise- and cold exposure-induced adaptation remains poorly understood. We therefore examined TSP1 regulation in greater detail across the datasets. Twenty-one genes showed consistent upregulation across all exercise types in the human exercise intervention, among which TSP1 was consistently upregulated across all exercise conditions (Fig. 1c). In the human cold-exposure dataset, cold exposure upregulated 1,316 genes in skeletal muscle (Fig. 1d), with TSP1 showing a significant increase (Fig. 1e). Our analysis identified 144 proteins that were significantly increased in the plasma of mice after 5 weeks of treadmill exercise training. Among them, TSP1 levels were elevated (Fig. 1f). The consistent upregulation of TSP1 in both skeletal muscle and plasma following exercise- and cold-induced contractions identified it as a primary candidate for further investigation.

### Exercise and cold exposure induce TSP1 release and proteolytic processing

**Fig. 2:**
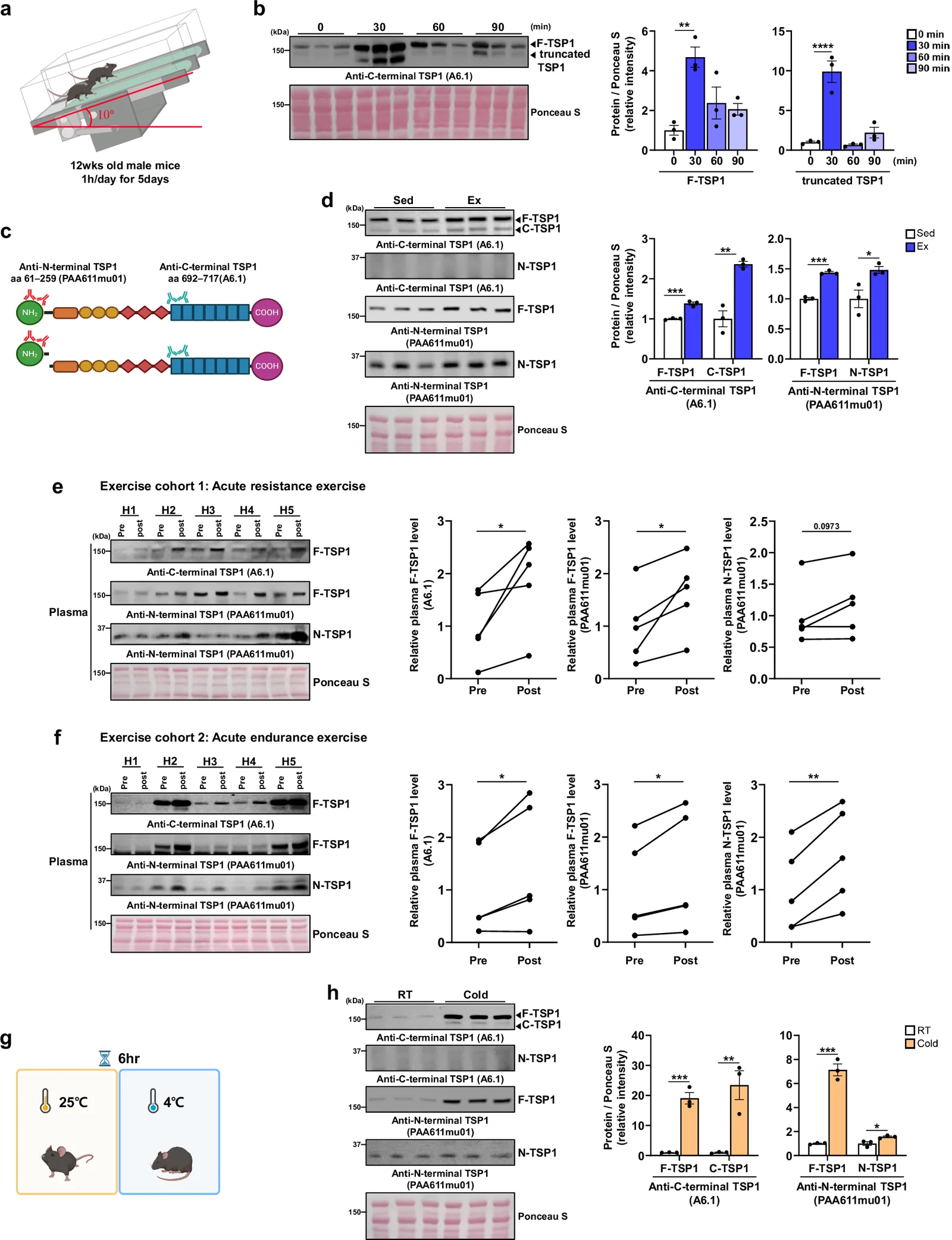
Plasma TSP1 levels are increased by exercise and cold exposure in vivo. **a** Schematic of the mouse exercise protocol. 12-week-old C57BL/6 J mice were acclimated to the treadmill over 5 days. Plasma samples were harvested after 0, 30, 60, and 90 min of acute treadmill exercise. **b** Western blots demonstrating expression of F-TSP1 and truncated TSP1 in plasma from exercised mice (left). Relative densitometric quantification of Western blots (right). **c** Schematic illustrating two TSP1 antibodies. **d** Immunoblots of F-TSP1 and N-TSP1 in plasma after 30 min of exercise (left). Relative densitometric quantification (*n* = 3 per group) (right). **e** Immunoblots of F-TSP1 and N-TSP1 in plasma from young acute resistance-exercised individuals (*n* = 5) (left). Comparisons between individual pre- and post-exercise protein levels of panel **e** are depicted in line graphs (*n* = 5) (right). **f** Immunoblots of F-TSP1 and N-TSP1 in plasma from young acute endurance-exercised individuals (*n* = 5) (left). Comparisons between individual pre- and post-exercise protein levels of panel **f** are depicted in line graphs (*n* = 5) (right). **g** Schematic of the cold-exposure experiment. **h** Immunoblots of F-TSP1 and N-TSP1 in plasma after cold exposure (left). Relative densitometric quantification (*n* = 3 per group) (right). Ponceau S staining was used as a loading control for immunoblot quantification in panels **b**, **d**, **e**, **f** and **h**. Data are presented as means ± SEM. *P* values were calculated using one-way ANOVA with Dunnett’s multiple comparison post hoc test (**b**), two-tailed unpaired Student’s *t* test (**d** and **h**) and two-tailed paired Student’s *t* test (**e** and **f**) following Shapiro–Wilk normality test. (*P < 0.05, **P < 0.01, ***P < 0.001, ****P < 0.0001). Cold, cold exposure (4°C); Ex, Exercise; RT, room temperature (25°C); Sed, Sedentary.

We first sought to verify the upregulation of TSP1 expression in muscles, other tissues and plasma in response to exercise or cold exposure using both in vitro and in vivo approaches. We investigated whether TSP1 expression was upregulated in C2C12 myotubes upon exposure to exercise-mimicking stimuli, including AICAR (an AMP-activated protein kinase activator), forskolin (an activator of adenylate cyclase) and ionomycin (a calcium ionophore). Our results showed that ionomycin-induced elevation of calcium levels led to increased expression of both TSP1 mRNA and protein in mouse C2C12 myotubes. In contrast, neither forskolin nor AICAR treatment resulted in significant changes in TSP1 expression levels (Supplementary Fig. 1a, b). We also verified that electrical pulse stimulation (EPS), used as an in vitro exercise model, enhanced TSP1 expression (Supplementary Fig. 1c). Next, we examined whether TSP1 expression increased in vitro under conditions that induce thermogenesis. Isoproterenol (ISO), a non-selective β-adrenergic agonist, is commonly used to mimic cold-induced adrenergic stimulation associated with BAT thermogenesis. Thus, we treated differentiated brown adipocytes with ISO and observed increased TSP1 expression (Supplementary Fig. 1d). To validate these findings in vivo, we assessed TSP1 expression in skeletal muscle and BAT, confirming its systemic upregulation in response to acute treadmill exercise. Mice were acclimated to treadmill running through a 5-day habituation period. Subsequently, they underwent 1-h acute treadmill exercise sessions with increasing intensity once daily for 5 consecutive days (Fig. 2a) (details provided in the Supplementary Information). Western blot analysis of plasma samples collected immediately after 0, 30, 60 and 90 min of treadmill exercise showed an increase in F-TSP1 levels in exercised mice compared with sedentary controls. Plasma TSP1 levels peaked at 30 min (Fig. 2b). Interestingly, we observed an approximately 120–140-kDa truncated TSP1. The proteolytic cleavage of TSP1 into fragments, leading to the local release of bioactive peptides, is well established as a key mechanism for regulating its activity^23^. To evaluate whether exercise upregulates plasma levels of TSP1 fragments, we performed Western blotting using different antibodies specific for the N- and C-terminal regions of TSP1 in plasma samples that were collected following 30 min of exercise with the same protocol described in Fig. 2a (Fig. 2c). To validate the specificity of our antibodies and explore TSP1 processing, we modulated TSP1 levels in C2C12 cells using ionomycin and siRNA. In conditioned media (CM), both ionomycin-induced upregulation and siRNA-mediated knockdown of TSP1 were clearly detected by anti-C-terminal (A6.1) and anti-N-terminal (PAA611mu01) antibodies. Notably, PAA611mu01 detected N-TSP1 in CM, whereas A6.1 did not, indicating epitope-specific detection. In contrast, N-TSP1 was not detected by PAA611mu01 in cell lysates, supporting the possibility that N-TSP1 is released following TSP1 secretion (Supplementary Fig. 2a, b). Collectively, these results confirm the functionality of the N-TSP1 antibody and support a model of extracellular proteolytic processing of TSP1. Consistent with these in vitro findings, exercise significantly increased both F-TSP1 and N-TSP1 levels in mouse plasma, indicating that exercise is associated with TSP1 proteolytic processing and release into the circulation. Notably, plasma N-TSP1 was detected exclusively by PAA611mu01, but not by A6.1 (Fig. 2d). Proteomic analysis revealed a significant increase in plasma TSP1 levels post-exercise, as shown in Fig. 1f. Subsequent analysis of peptide intensities revealed no significant differences among plasma N-TSP1, M-TSP1 and C-TSP1 levels after exercise (Supplementary Fig. 3) (details provided in the Supplementary Information). Given that TSP1-related fragments were altered in mouse plasma following exercise, we next investigated whether similar changes occur in humans. Blood samples were collected from young participants before and after acute resistance or endurance exercise (details provided in the Supplementary Information). Both exercise interventions significantly increased plasma F-TSP1 levels, as detected by both A6.1 and PAA611mu01 antibodies. By comparison, plasma N-TSP1 levels showed a differential response to exercise type, with a modest increase after acute resistance exercise and a significant elevation after acute endurance exercise (Fig. 2e, f). Next, we investigated whether cold exposure would induce similar effects on TSP1 expression as observed with exercise. To assess this hypothesis, mice were exposed to 4°C for 6 h (Fig. 2g). Cold exposure elevated plasma levels of F-TSP1, C-TSP1 and N-TSP1 compared with those at 25°C (Fig. 2h). Collectively, these findings demonstrate that both exercise and cold exposure promote TSP1 secretion and are associated with the generation of circulating N-TSP1.

### Exercise and cold exposure upregulate TSP1 expression in skeletal muscle and brown adipose tissue

**Fig. 3:**
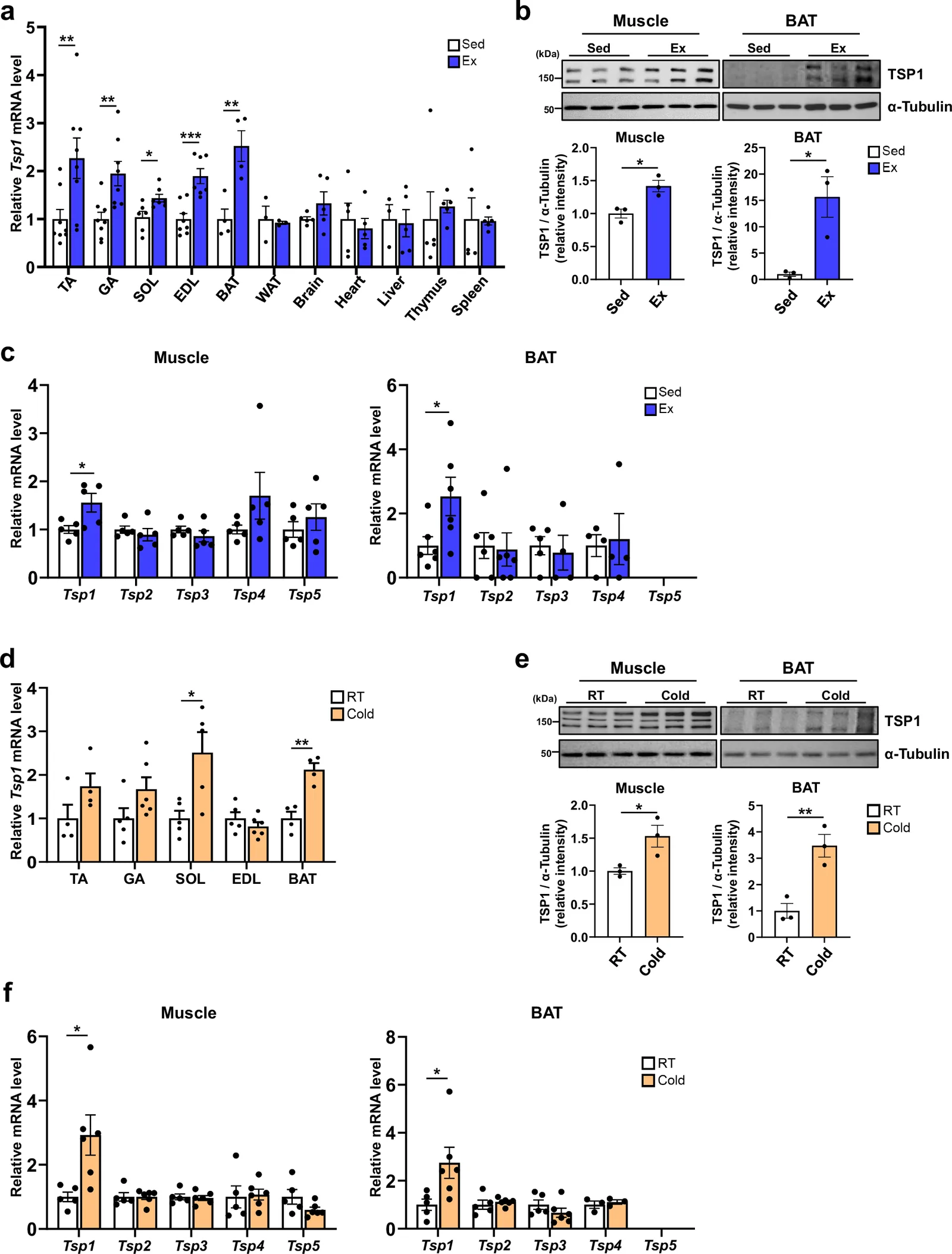
TSP1 levels are elevated in soleus muscle and brown adipose tissue in response to exercise and cold-exposure stimuli in vivo. **a** *Tsp1* mRNA expression in skeletal muscle and other tissues following exercise (*n* = 4–8 per group). **b** TSP1 immunoblots in soleus muscle (SOL) and brown adipose tissue (BAT) after 30 min of exercise. α-Tubulin was used as a loading control (upper). Relative densitometric quantification of TSP1 protein levels (*n* = 3 per group) (lower). **c** mRNA expression levels *of Tsp1, Tsp2, Tsp3, Tsp4* and *Tsp5* in SOL (left) and BAT (right) after treadmill exercise. **d** *Tsp1* mRNA expression in skeletal muscle and BAT after cold exposure (*n* = 4–6 per group). **e** TSP1 immunoblots in SOL and BAT after cold exposure. α-Tubulin was used as a loading control (upper). Relative densitometric quantification of TSP1 protein levels (*n* = 3 per group) (lower). **f** mRNA expression levels of *Tsp1, Tsp2, Tsp3, Tsp4* and *Tsp5* in SOL (left) and BAT (right) after cold-exposure stimuli (*n* = 4–6 per group). Data are presented as mean ± SEM. *P* values were calculated using two-tailed unpaired Student’s *t* test (**a–f**) following Shapiro–Wilk normality test. Mann–Whitney *U* test was employed for non-parametric data (**a**, **c** and **f**). *P < 0.05, **P < 0.01, ***P < 0.001. Cold, cold exposure (4°C); EDL, extensor digitorum longus; Ex, Exercise; GA, gastrocnemius; RT, room temperature (25°C); Sed, Sedentary; TA, tibialis anterior.

TSP1 is a secreted matricellular glycoprotein expressed in various cell types, including platelets, endothelial cells, immune cells and muscle cells^24–27^. It circulates at low concentrations in plasma and is responsive to physiological stresses. To determine whether exercise and cold exposure regulate TSP1 expression, we analysed its mRNA and protein levels in multiple tissues. *Tsp1* mRNA levels increased immediately after 30 min of exercise in skeletal muscles, including the tibialis anterior (TA), gastrocnemius, soleus, extensor digitorum longus and BAT, whereas no significant changes were detected in other tissues, including WAT, brain, heart, liver, thymus and spleen (Fig. 3a). Further analysis by Western blotting confirmed a significant increase in TSP1 protein expression in both muscle and BAT following exercise (Fig. 3b). Exercise did not alter the expression levels of *Tsp2*, *Tsp3*, *Tsp4* and *Tsp5* in muscle and BAT (Fig. 3c). We next investigated whether cold exposure also affects TSP1 expression in mice. Cold exposure induced a significant upregulation of *Tsp1* mRNA expression in skeletal muscle and BAT (Fig. 3d), accompanied by elevated TSP1 protein levels (Fig. 3e). Consistent with exercise results, cold exposure selectively increased TSP1 expression, whereas other TSP family members remained unchanged (Fig. 3f). Our findings indicate that exercise and cold exposure specifically upregulate TSP1 expression in vivo.

### N-terminal TSP1 fragment exhibits bioactivity on brown adipocytes and myotubes in vitro

**Fig. 4:**
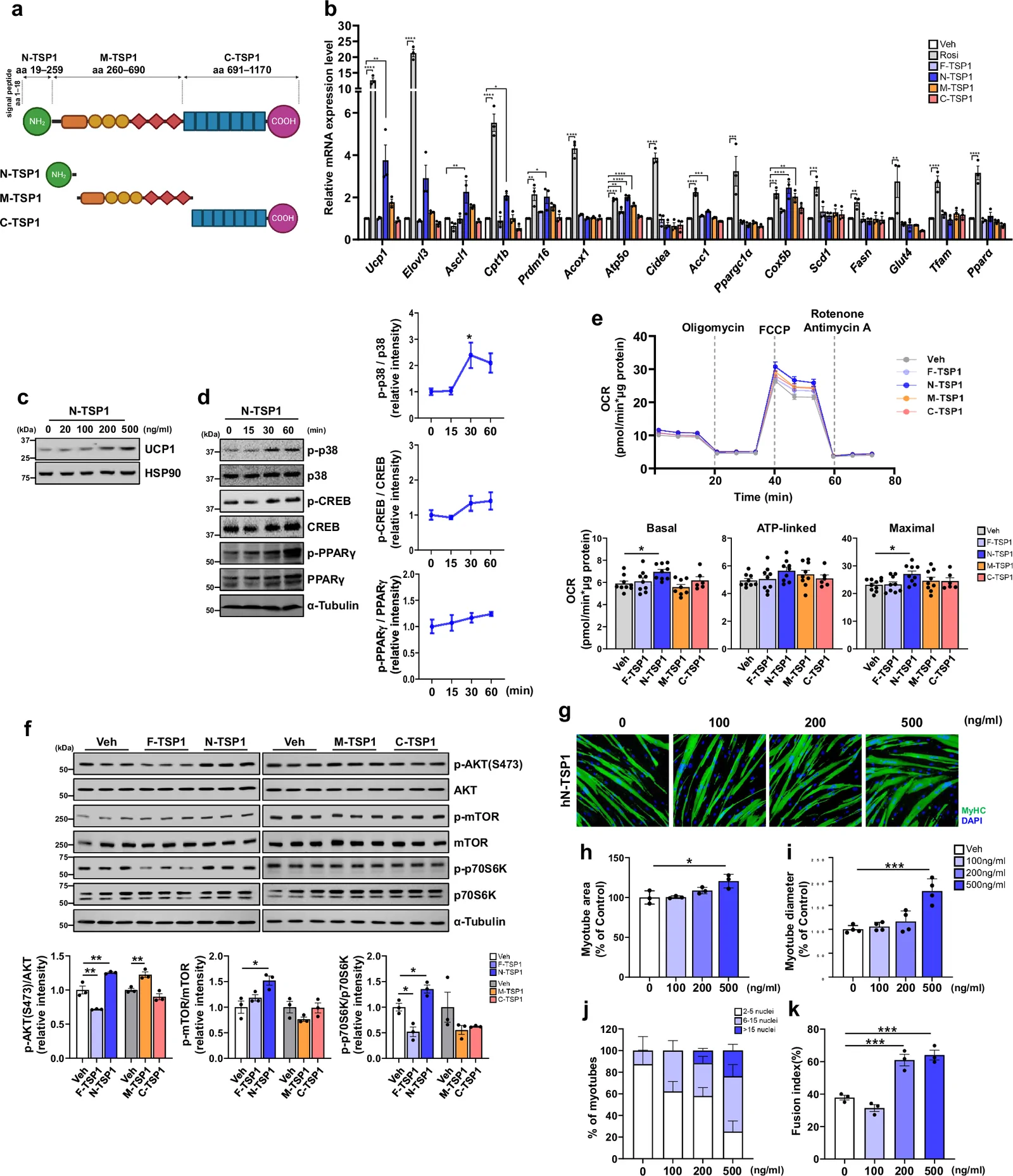
N-TSP1 treatment increases the expression of thermogenesis-related genes in adipocytes and mitochondrial respiration in myotubes*.* **a** Representative schematic of F-TSP1 showing the three different fragments: N-TSP1 (aa 19–259), M-TSP1 (aa 260–690), C-TSP1 (aa 691–1170) (upper). Schematic illustration of TSP1 cleavage products (lower). **b** Relative mRNA expression of thermogenesis-related genes in differentiated stromal vascular fraction adipocytes treated with F-TSP1 and three TSP1 fragments (1 μg/ml) for 24 h. Rosiglitazone (1 μM) served as a positive control (*n* = 3 per group). **c** Western blot showing the dose-dependent effect of N-TSP1 on UCP1 protein levels. **d** Western blot analysis of expression and phosphorylation levels of p38, CREB and PPARγ in differentiated brown adipocytes treated with N-TSP1 (500 ng/ml) (left). Quantification of protein levels (right). **e** Oxygen consumption rate was measured using extracellular flux analysis in C2C12 myotubes differentiated in medium containing 1 μg/ml of F-TSP1, N-TSP1, M-TSP1 or C-TSP1, starting 1 day after differentiation. Medium was changed daily for 3 consecutive days (upper). Measurements of basal respiration, ATP production and maximal respiration (*n* = 6–10 per group). Respiration data were normalized by protein concentration (lower). **f** Western blot analysis of phosphorylated AKT-mTOR signalling pathway proteins in differentiated C2C12 myotubes after 24-h treatment with F-TSP1, N-TSP1, M-TSP1 or C-TSP1 (1 μg/ml) (upper). Relative densitometric quantification (*n* = 3 per group) (lower). **g** Myosin heavy chain (MyHC) was immunostained in human myotubes after 3 days of differentiation with human N-TSP1 (hN-TSP1). **h–k** Analysis of differentiated human myotube area (*n* = 3 per group) (**h**), diameter (*n* = 4 per group) (**i**), percentage of myotubes with different nuclei numbers (2–5, 6–15 or >15 nuclei) (*n* = 3 per group) (**j**), fusion index, defined as the percentage of multinucleated myotubes per field (*n* = 3 per group) (**k**). Data are presented as means ± SEM. *P* values were calculated using one-way ANOVA with Dunnett’s multiple comparison post hoc test (**b**, **d**, **e**, **f**, **h**, **i** and **k**) following Shapiro–Wilk normality test. *P < 0.05, **P < 0.01, ***P < 0.001, ****P < 0.0001.

Given that exercise and cold exposure promote TSP1 secretion and fragment release in skeletal muscle and BAT, we next sought to identify which TSP1 fragments underlie its bioactivity. Previous studies have suggested that TSP1 fragments may possess distinct biological functions^23^. To explore this possibility, we generated N-TSP1 (aa 19–259, excluding the signal peptide), M-TSP1 (aa 260–690) and C-TSP1 (aa 691–1170) (Fig. 4a). To evaluate their potential effects on thermogenic activation, we utilized primary stromal vascular fraction (SVF) cells isolated from mouse WAT, which retains the capacity to differentiate into beige adipocytes. Primary SVF cells were differentiated into brown-like adipocytes over a period of 7 days and subsequently treated with full-length TSP1 and its fragments for 24 h. Under these screening conditions, N-TSP1 treatment significantly upregulated thermogenic (*Ucp1*, *Ascl1*, *Prdm16*), fatty acid oxidation (*Cpt1b*, *Acc1*) and mitochondrial oxidative phosphorylation-related genes (*Atp5o*, *Cox5b*) in differentiated SVF adipocytes (Fig. 4b). N-TSP1 also increased the expression of UCP1 in a dose-dependent manner (Fig. 4c) and rapidly and transiently activated the p38 signalling pathway in differentiated brown adipocytes (Fig. 4d), which serves as a central regulator of cyclic AMP (cAMP)-dependent *Ucp1* transcription in brown adipocytes^28^.

Given that various myokines released during muscular contractions are well known for their beneficial effects on muscle function^29^, we next investigated whether N-TSP1 affects muscle-cell function. Oxygen consumption rate (OCR) analysis in C2C12 myotubes revealed that N-TSP1 exclusively increased both basal and maximal respiration after 48 h of treatment, whereas other fragments had no effect (Fig. 4e). In addition, N-TSP1 activated protein-synthesis signalling, as indicated by elevated phosphorylation of AKT and p70S6K, whereas M-TSP1 also increased AKT phosphorylation and F-TSP1 reduced phosphorylation of these kinases (Fig. 4f). In line with these findings, N-TSP1 treatment promoted myotube hypertrophy during myogenic differentiation of human primary myoblasts, as indicated by an increased number of nuclei per myotube and elevated fusion index (Fig. 4g–k). Moreover, N-TSP1 dose-dependently increased myotube area and diameter in C2C12 myoblasts and enhanced protein synthesis, as confirmed by a puromycin incorporation assay (Supplementary Fig. 4a, b). N-TSP1 treatment did not alter cell viability or proliferation (Supplementary Fig. 4c, d). Collectively, these results demonstrate that N-TSP1 may play a role in promoting thermogenesis in adipocytes and enhancing both protein synthesis and mitochondrial respiration in muscle cells.

### N-TSP1 ameliorates metabolic dysfunction in a high-fat-diet mouse model

**Fig. 5:**
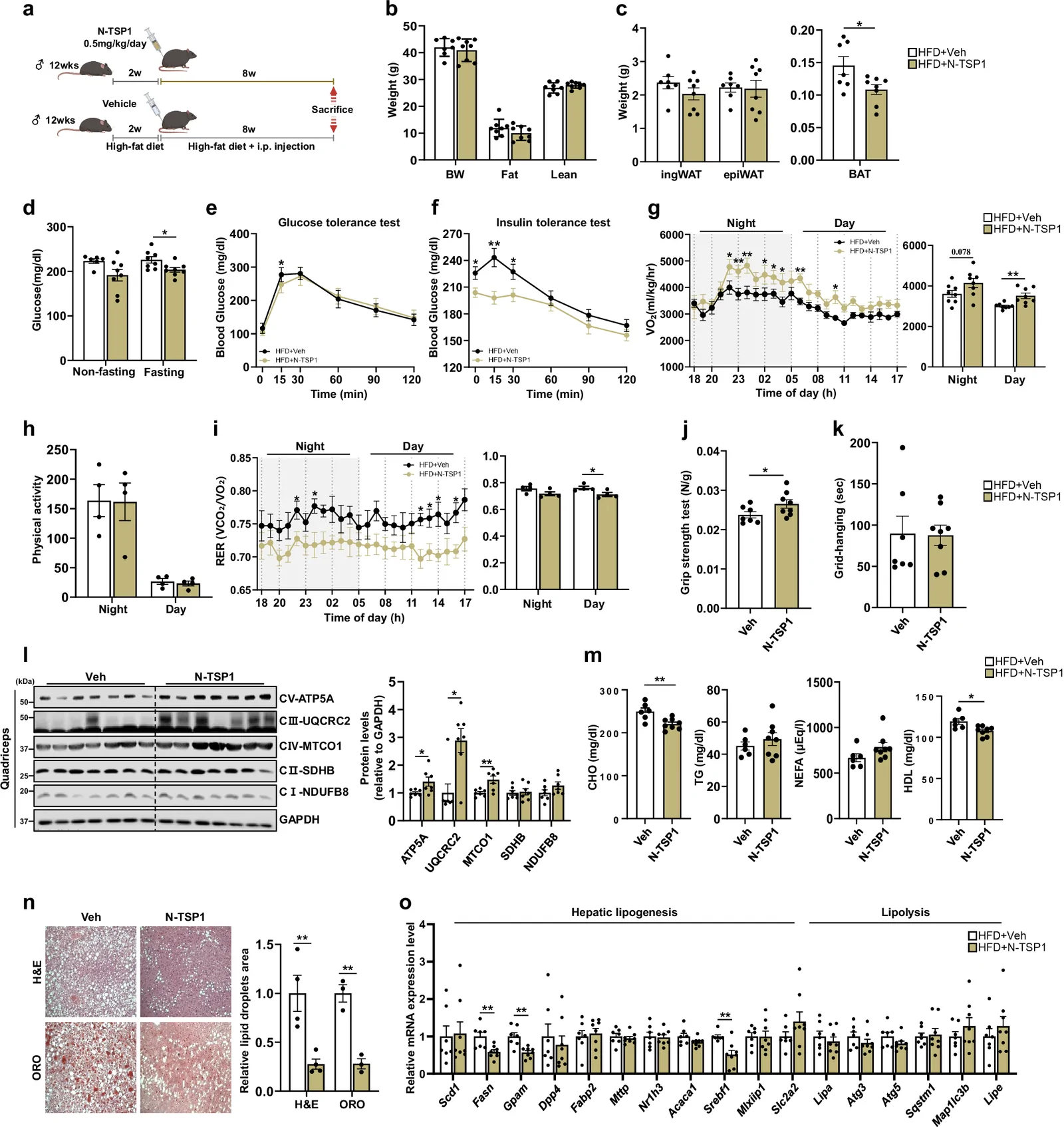
N-TSP1 treatment enhances skeletal muscle function and improves lipid metabolism in obese mice. **a** Schematic showing high-fat-diet (HFD) duration and vehicle or N-TSP1 supplementation. 12-week-old mice were fed HFD for 2 weeks prior to N-TSP1 intraperitoneal (i.p.) injection (0.5 mg/kg/day) for 8 weeks (*n* = 7–8 per group). **b** Body weight (BW) and total fat (Fat) or lean mass (Lean) in HFD-fed mice treated with vehicle or N-TSP1 (*n* = 7–8 per group). **c** Weight of inguinal white adipose tissue (ingWAT) and epididymal white adipose tissue (epiWAT) (left) and brown adipose tissue (BAT) (right) (*n* = 7–8 per group). **d** Blood glucose levels under non-fasting and 6-h fasting conditions (*n* = 8 per group). **e** Glucose tolerance test (GTT) after a 12-h fast in HFD-fed mice, with glucose administered i.p. (*n* = 8 per group). **f** Insulin tolerance test (ITT) after a 6-h fast in HFD-fed mice, with insulin administered i.p. (*n* = 8 per group). **g** Oxygen consumption (VO_2_) of HFD-fed mice treated with vehicle or N-TSP1 over 24 h after 8 weeks of supplementation (left). Average night- and day-time oxygen consumption rate (*n* = 8 per group) (right). **h** Average night- and day-time activity is shown (*n* = 4 per group). **i** Respiratory exchange ratio (RER) of HFD groups treated with vehicle or N-TSP1 over 24 h after 8 weeks of supplementation (left). Average RER during light and dark cycles (right). **j** Hindlimb strength (Grip strength test) measurement. **k** Grid-hanging time measurement. **l** Western blot analysis of mitochondrial oxidative phosphorylation (OXPHOS) complexes in quadriceps muscle (left). Relative densitometric quantification was normalized by GAPDH values (*n* = 7 per group) (right). **m** Plasma levels of cholesterol (CHO), triglyceride (TG), non-esterified fatty acid (NEFA) and HDL (*n* = 6–8 per group). **n** Haematoxylin and eosin staining (H&E) and Oil red O (ORO) staining of hepatic sections from mice treated with vehicle or N-TSP1 (left). Quantification of lipid droplet area (*n* = 7–8 per group) (right). **o** Relative mRNA expression of steatosis-related genes by quantitative PCR. Data are presented as means ± SEM. *P* values were calculated using two-tailed unpaired Student’s *t* test (**b**, **c**, **d**, **g**, **h**, **i**, **j**, **k**, **l**, **m**, **n** and **o**) and two-way repeated-measures ANOVA with Tukey’s multiple comparison post hoc test (**e**, **f**, **g** and **i**) following the Shapiro–Wilk normality test. Mann–Whitney *U* test was employed for non-parametric data (**o**). *P < 0.05, **P < 0.01.

Based on our in vitro findings in myotubes and adipocytes, we hypothesized that N-TSP1 would improve metabolic homeostasis in HFD-fed mice. To test this, C57BL/6J male mice were fed a HFD for 2 weeks, followed by daily intraperitoneal injections of N-TSP1 for 8 weeks while maintaining the HFD (Fig. 5a). There were no significant differences in BW, total fat (Fat), and lean mass (Lean) between vehicle- and N-TSP1-injected groups (Fig. 5b). N-TSP1 injection did not affect inguinal WAT (ingWAT) or epididymal WAT weight, whereas BAT weight was significantly lower in N-TSP1-injected mice (Fig. 5c). Blood glucose levels in the non-fasted state were comparable between N-TSP1- and vehicle-injected mice. In contrast, under fasting conditions, N-TSP1-injected mice exhibited significantly lower blood glucose levels than the vehicle-treated group (Fig. 5d). Notably, N-TSP1-injected mice exhibited improved glucose tolerance during the glucose tolerance test and enhanced insulin sensitivity during the insulin tolerance test (ITT) (Fig. 5e, f). Energy expenditure was significantly higher in the N-TSP1 group (Fig. 5g) without changes in locomotor activity or food intake (Fig. 5h, Supplementary Fig. 5a). The respiratory exchange ratio was reduced, indicating enhanced lipid oxidation, probably reflecting increased adipose-derived substrate utilization (Fig. 5i). N-TSP1-treated mice also exhibited a transient rise in heat production during the dark phase, whereas average heat production at ambient temperature and rectal temperature at 25°C and 18°C remained unchanged (Supplementary Fig. 5b, c).

Because N-TSP1 activated p38 signalling in differentiated brown adipocytes (Fig. 4d), we examined this pathway in vivo. N-TSP1 treatment increased p38 phosphorylation in BAT, whereas phosphorylation of CREB and PPARγ and the expression of UCP1 were unchanged (Supplementary Fig. 6a). Similarly, browning-related markers (UCP1, PRDM16 and PPARγ) in WAT were unaffected (Supplementary Fig. 6b). To further explore the systemic effects of N-TSP1, we next analysed skeletal muscle from HFD-fed mice injected with N-TSP1. Grip strength was significantly greater in N-TSP1-treated mice than in vehicle-treated controls (Fig. 5j), whereas grid hanging time and muscle mass were comparable between groups (Fig. 5k, Supplementary Fig. 7a). N-TSP1 administration upregulated OXPHOS complex proteins ATP5A, UQCRC2 and MTCO1 in muscle (Fig. 5l) and increased phosphorylation of p70S6K in skeletal muscle tissue (Supplementary Fig. 7b). Plasma biochemical analyses revealed that N-TSP1 supplementation reduced total cholesterol and HDL cholesterol levels, whereas triglyceride and non-esterified fatty acid levels were unchanged (Fig. 5m). There were no significant differences between the groups in other blood parameters, including LDL cholesterol, aspartate aminotransferase (AST), and alanine aminotransferase (ALT) (Supplementary Fig. 8a–k). Histological analysis revealed that N-TSP1 markedly reduced hepatic lipid accumulation compared with vehicle-treated mice, as shown by haematoxylin and eosin and Oil Red O staining (Fig. 5n). Consistent with this, *Fasn, Gpam* and *Srebf1* expression was decreased in the liver of HFD mice, whereas expression of lipolysis-related genes remained unchanged (Fig. 5o). However, N-TSP1 did not significantly alter lipid accumulation in HepG2 cells (Supplementary Fig. 9), suggesting that the hepatic effects of N-TSP1 observed in vivo may involve systemic or indirect mechanisms.

Next, we examined the effects of N-TSP1 injection in mice fed a standard diet (Supplementary Fig. 10a). There were no significant differences in BW, muscle mass, fat mass, or running performance between N-TSP1-treated and vehicle-treated groups. Notably, N-TSP1-treated mice exhibited increased grip strength compared with vehicle-treated mice (Supplementary Fig. 10b–g). Consistent with the findings from HFD-fed mice, we observed increased expression of ATP5A, UQCRC2 and SDHB, as well as elevated phosphorylation of p70S6K (Supplementary Fig. 10h, i). These molecular changes may underlie the enhanced muscle strength by promoting protein synthesis and mitochondrial function.

As part of baseline metabolic characterization, we assessed glucose and insulin tolerance in young mice (Supplementary Fig. 11a). N-TSP1 treatment had no significant effect on glucose tolerance. During the ITT, however, blood glucose levels were higher in N-TSP1-treated mice at 15 and 60 min after insulin administration, suggesting a transient difference in insulin-induced glucose lowering under basal conditions (Supplementary Fig. 11b). N-TSP1 increased energy expenditure during the light phase but not the dark phase, indicating a mild elevation of resting energy expenditure under normal chow diet conditions (Supplementary Fig. 11c). Heat production and respiratory exchange ratio remained unchanged (Supplementary Fig. 11d, e). Biochemical analyses revealed no significant differences in plasma cholesterol, HDL, triglyceride, non-esterified fatty acid or LDL levels between the two groups (Supplementary Fig. 11f). In BAT, phosphorylation of PPARγ at Ser273 was decreased, and UCP1 expression was increased in N-TSP1-treated mice compared with vehicle-treated controls. In ingWAT, UCP1 and PPARγ levels remained unchanged, whereas PRDM16 levels were increased (Supplementary Fig. 11g, h). This marker pattern suggests limited changes in adipose thermogenic remodelling under basal young conditions.

Taken together, our data indicate that N-TSP1 improves metabolic parameters in diet-induced obesity and modestly enhances energy expenditure, even under normal dietary conditions. These findings imply that N-TSP1 acts on both skeletal muscle and BAT to modulate whole-body energy balance and muscle function.

### N-TSP1 improves muscle function and promotes metabolic remodelling in aged mice

**Fig. 6:**
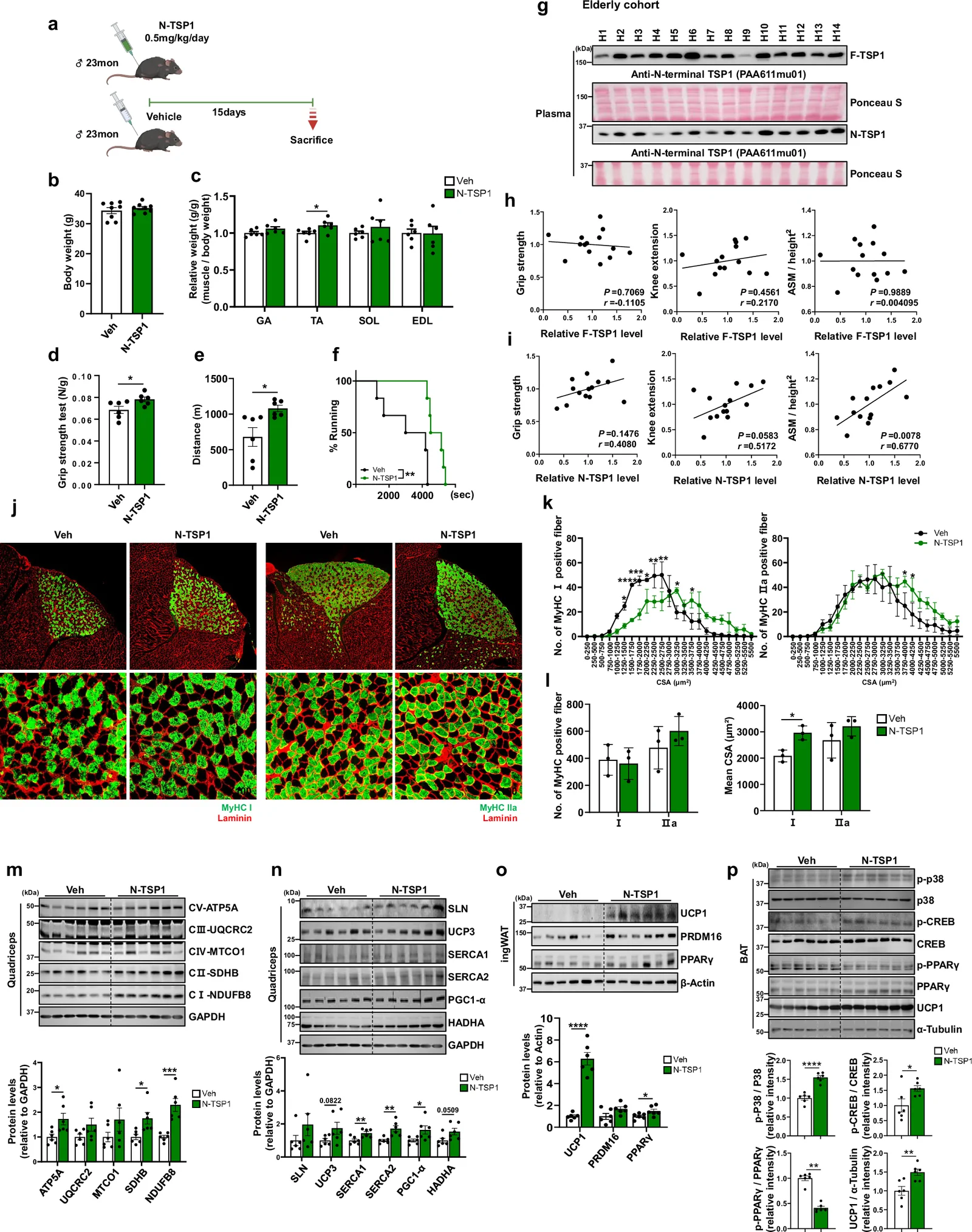
N-TSP1 administration improves muscle function and promotes metabolic remodelling in aged mice. **a** Schematic showing N-TSP1 supplementation. 23-month-old mice received vehicle or N-TSP1 (0.5 mg/kg/day) intraperitoneal injection for 15 days. **b** Body weight measurement (n = 8 per group). **c** Muscle mass (*n* = 6 per group). **d–f** Physical performance tests: grip strength (**d**), distance to exhaustion (**e**), and time to exhaustion (**f**). **g** Immunoblots showing F-TSP1 and N-TSP1 levels in plasma of elderly individuals (*n* = 14). **h**–**i** Correlation scatter plots between plasma protein levels and parameters of body composition and physical performance (grip strength, knee extension and appendicular skeletal muscle (ASM)/height^2^). **h** Correlation scatter plots between F-TSP1 plasma levels and parameters (*n* = 14). **i** Correlation scatter plots between N-TSP1 plasma levels and parameters (*n* = 14). **j** Immunostaining with myosin heavy chain (MyHC I) (green) (left), MyHC IIa (green) (right) and laminin (red) in the soleus muscle of vehicle (Veh) and N-TSP1-injected aged mice. **k**, **l** Muscle fibre size and distribution. k, Frequency distribution of MyHC I-positive fibre cross-sectional areas (CSA) (left) and MyHC IIa fibre CSA (right). **l** Comparison of MyHC positive fibre number (left) and average CSA (right) (*n* = 3 per group). **m** Immunoblots of mitochondrial OXPHOS complexes in the quadriceps muscle of aged mice (upper). Relative densitometric quantification was normalized by GAPDH values (lower) (*n* = 6 per group). **n** Immunoblots of proteins associated with oxidative metabolism and muscle-related thermogenic energy utilization in quadriceps muscle of aged mice (upper). Relative densitometric quantification was normalized by GAPDH values (lower) (*n* = 6 per group). **o** Immunoblots showing expression of UCP1, PRDM16 and PPARγ in ingWAT of aged mice (upper). Relative densitometric quantification was normalized by β-actin values (*n* = 6 per group) (lower). **p** Immunoblots of expression and phosphorylation levels of p38, CREB, PPARγ and UCP1 in brown adipose tissue (BAT) of aged mice (upper). Relative densitometric quantification (lower) (*n* = 6 per group). Data are presented as means ± SEM. *P* values were calculated using two-tailed unpaired Student’s *t* test (**b**, **c**, **d**, **e**, **l**, **m**, **n**, **o**, and **p**), two-way repeated-measures ANOVA with Tukey’s multiple comparison post hoc test (**k**) and Pearson’s correlation coefficient (*r*) was employed in panels **h** and **i** to assess correlations and *P* values following the Shapiro–Wilk normality test. Mann–Whitney *U* test was employed for non-parametric data (**l** and **p**). Log-rank (Mantel–Cox) test was employed to calculate the *P* value in panel **f**. *P < 0.05, **P < 0.01, ***P < 0.001, ****P < 0.0001. EDL, extensor digitorum longus; GA, gastrocnemius; SOL, soleus; TA, tibialis anterior.

Given the well-established link between ageing and the development of metabolic abnormalities and muscle weakness, we sought to determine whether N-TSP1 treatment could ameliorate age-related declines in vivo. Aged mice received daily intraperitoneal injections of vehicle or N-TSP1 (0.5 mg/kg) for 15 days (Fig. 6a). N-TSP1 treatment led to a significant increase in TA muscle mass, whereas overall BW and the mass of other muscles remained unchanged (Fig. 6b, c). N-TSP1-injected aged mice exhibited improved physical performance, as evidenced by increased grip strength and enhanced treadmill endurance (Fig. 6d–f). To explore the clinical relevance of these findings, we analysed correlations between circulating TSP1 levels and physical-performance parameters in an elderly cohort. Plasma F-TSP1 levels showed no significant correlation with either grip strength or knee extension ability. In contrast, N-TSP1 levels exhibited a positive association with both parameters, as well as with appendicular skeletal muscle mass normalized to height (ASM/height^2^) (Fig. 6g–i). In the soleus muscle, N-TSP1 increased the cross-sectional area of MyHC I-positive muscle fibres without altering fibre-type distribution (Fig. 6j–l). Similarly, in the TA muscle, the cross-sectional area of both MyHC IIa- and IIb-positive muscle fibres was increased without changes in fibre type composition (Supplementary Fig. 12a). Notably, despite no detectable changes in markers of protein synthesis signalling (Supplementary Fig. 12b), N-TSP1 administration elevated the expression of genes involved in mitochondrial biogenesis (*Ppargc1α* and *Tfam*), oxidative phosphorylation (*Sdhb* and *Atp5b*), β-oxidation (*Acox1* and *Pparα*), glucose uptake (*Glut4*), and muscle fibre-type specification (*Nfatc3*) (Supplementary Fig. 12c) and increased OXPHOS complex protein levels (Fig. 6m). As N-TSP1 improved muscle function and enhanced mitochondrial-related markers in aged skeletal muscle, we further examined whether these changes were accompanied by molecular signatures associated with metabolic adaptation. In skeletal muscle, N-TSP1 treatment increased or tended to increase the mRNA and protein expression of factors related to oxidative metabolism and muscle energy utilization, including sarcolipin, UCP3, SERCA1/2, PGC-1α and HADHA (Fig. 6n, Supplementary Fig. 12d). Consistent with tissue-specific metabolic adaptation, browning-associated markers, including UCP1 and PPARγ, were significantly upregulated in ingWAT of N-TSP1-treated mice (Fig. 6o). In BAT, N-TSP1 increased phosphorylation of p38 and CREB, upregulated UCP1 and reduced PPARγ Ser273 phosphorylation (Fig. 6p). Because therapeutic strategies targeting PPARγ modulation in type 2 diabetes have focused on reducing Ser273 phosphorylation to achieve partial agonism of the receptor^30^, these findings suggest that N-TSP1 may improve glucose metabolism, at least in part, by suppressing PPARγ Ser273 phosphorylation. Collectively, these findings suggest that N-TSP1 improves muscle function in aged mice, accompanied by changes in mitochondrial and oxidative metabolic markers in skeletal muscle and molecular adaptations in ingWAT and BAT.

## Discussion

TSPs are tissue- and context-specific matricellular proteins that interact with receptors, growth factors and extracellular matrix proteins^23^. TSP1 plays a multifaceted role in various biological processes, including angiogenesis^31^, inflammatory responses^32^, tumour growth^33^, fibrotic events^34^ and ageing^35^. Notably, the translational path for TSP1-based therapies is informed by prior clinical experience with TSP1-derived mimetic peptides developed as anti-angiogenic agents. Their anti-angiogenic activity is mediated, at least in part, through interactions between the type 1 repeat domains of TSP1 and CD36. Among these peptides, ABT-510, which is derived from the second type 1 repeat domain of TSP1, advanced to phase I/II clinical trials for advanced cancers, whereas related analogues, including ABT-898, have been explored in preclinical disease models^36–38^. Although these studies demonstrated that TSP1-derived anti-angiogenic approaches were generally well tolerated, their therapeutic efficacy in solid tumours was modest. It is important to note, however, that the biological axis identified in the present study, centred on N-TSP1, is distinct from these earlier anti-angiogenic strategies and is instead associated with musculoskeletal and metabolic adaptation. This functional divergence suggests that N-TSP1 may offer a unique therapeutic window that is independent of the challenges encountered in earlier TSP1-based anticancer strategies.

We found that muscles stimulated by exercise and cold exposure secrete TSP1, and that truncated N-TSP1 plays a role in skeletal muscle and adipose tissues; however, we did not identify the protease that cleaves TSP1 during exercise and cold exposure. TSP1 is cleaved into N-terminal and C-terminal fragments by proteases such as neutrophil elastase (ELANE), HtrA1, thrombin, cathepsin G, ADAMTS7 and ADAMTS1 (refs.^15,39,40^). We investigated whether exercise influences the expression of several proteases in skeletal muscle. Exercise specifically upregulated *Elane* mRNA expression (Supplementary Fig. 13a, b), which is consistent with previous reports demonstrating neutrophil recruitment to muscle tissue during exercise^41,42^. It has been demonstrated that ELANE cleaves F-TSP1 protein into C-TSP1 and N-TSP1 (25 kDa) fragments in vitro^40^. However, further research is needed to identify the specific protease and amino acid cleavage sites responsible for N-TSP1 generation during exercise and cold exposure. These findings suggest that protease-mediated regulation of TSP1 in response to exercise and cold exposure is a critical event in determining the physiological effects of TSP1.

Studies using TSP1-null mice revealed its critical role as an endogenous inhibitor of angiogenesis, thereby preventing excessive blood vessel formation in the heart and skeletal muscles^43^. These mice are protected from both HFD-induced insulin resistance and fibrotic remodelling of skeletal muscle tissue^44^. Knockout mouse models provide insight into TSP1 function but are limited in capturing its full complexity, as proteolytic cleavage produces fragments with distinct functions. For instance, the N-terminal fragment promotes tube formation, whereas full-length TSP1 inhibits it^15^. In addition, N-TSP1, but not C-TSP1, increases endothelial cell invasiveness^45^. In a similar manner, our findings revealed that, compared with other TSP1 fragments and F-TSP1, N-TSP1 preferentially promoted a thermogenic marker signature in adipocytes and enhanced mitochondrial respiration in skeletal muscle myotubes. TSP1 is a trimeric extracellular matrix protein linked by disulfide bonds that interacts with various proteins. Therefore, cleaving TSP1 disrupts its three-dimensional conformation, thereby affecting the accessibility of binding domains and interactions with other molecules. Indeed, intact TSP1 binds to heparin more strongly (Kd = 41 nM) than the isolated N-terminal domain obtained through trypsin digestion (Kd = ~850 nM)^46^.

The mechanisms by which N-TSP1 interacts with other molecules to exert its functions are not fully understood. Of note, N-TSP1 interacts with integrins (α3β1, α4β1, α6β1 and α9β1)^47^. Integrin α6β1 is essential for proper muscle fibre organization in mice and zebrafish^48^. In addition, Ruiz-Ojeda et al. identified integrin–extracellular matrix interactions as crucial regulators of WAT and BAT function. Their study showed that loss of β1 integrin activity leads to lipodystrophy and systemic insulin resistance^49^. Although N-TSP1 interacts with integrins, the specific receptors that mediate its function in skeletal muscle and adipose tissue are unknown. We examined whether N-TSP1 treatment could activate the integrin signalling pathway, specifically focusing on focal adhesion kinase (FAK) and Src phosphorylation, in C2C12 myotubes. Integrin signalling is primarily mediated by intracellular signalling molecules such as FAK and members of the Src-family of protein tyrosine kinases^50^. However, no significant differences in FAK and Src phosphorylation were observed compared with the control group (Supplementary Fig. 14). The N-terminal domain of TSP1 binds to a wide range of cell-surface molecules beyond integrins, including heparin, LRP1/calreticulin, TSG6, aggrecan, decorin and sulphated glycolipids^47^. Therefore, further research is needed to unravel the mechanisms by which N-TSP1 interacts with these cell-surface molecules to exert its effects on skeletal muscle and adipose tissue.

Although our data highlight the metabolic benefits of N-TSP1, it is essential to consider these findings in the context of the canonical TSP1–CD47 signalling axis. Extensive literature, including recent evidence that CD47 blockade can mitigate obesity-related metabolic dysfunction^51^ and that targeting the TSP1/CD47 interface has antithrombotic potential^52^, underscores the detrimental role of full-length TSP1 in inhibiting nitric oxide signalling and vascular perfusion. However, N-TSP1 is a truncated fragment lacking the C-terminal CD47-binding domain, suggesting a mechanistic divergence. We hypothesize that N-TSP1 exerts its beneficial effects on muscle and systemic metabolism via a distinct, yet-to-be-identified receptor, potentially circumventing the vasoinhibitory trade-offs associated with the full-length protein. Nevertheless, further investigation into the receptor specificity of N-TSP1 is required to ensure that therapeutic elevation of this fragment does not inadvertently engage CD47-mediated pathways in high-risk vascular contexts.

Platelets play a crucial role in haemostasis and cancer metastasis and were the original source from which TSP1 was isolated^53^. TSP1, a major component of platelet α-granules, is rapidly released upon platelet activation. Vigorous exercise is known to induce platelet activation and aggregation, resulting in increased thrombin and fibrin formation^54,55^. Similarly, exposure to low ambient temperatures has been associated with enhanced platelet activation^56^. Although our study provides strong evidence that muscle and BAT are primary sources of TSP1 secretion during exercise and cold exposure, the contribution of platelets to circulating TSP1 levels cannot be overlooked. Further investigation is warranted to elucidate the relative contributions of muscle, BAT and platelets to the observed increase in circulating TSP1 following exercise and cold exposure.

Although N-TSP1 elicits distinct responses in muscle and adipose tissue, these tissue-specific responses appear to converge on mitochondrial and metabolic regulatory pathways associated with exercise and cold exposure. Adipose thermogenesis depends on UCP1, which is located in the mitochondrial inner membrane. UCP1 dissipates the proton gradient generated by the electron transport chain as heat rather than producing ATP^57^. In contrast, skeletal muscle mitochondrial respiration utilizes this proton gradient to synthesize ATP via ATP synthase. While both processes rely on the electron transport chain, they serve different purposes: heat generation in adipose tissue versus ATP production in muscle tissue. These processes are interconnected through shared regulatory pathways, including PGC-1α, AMPK signalling, fatty acid oxidation and mitochondrial dynamics^58,59^. Unlike canonical thermogenic factors that consistently elevate energy expenditure across various physiological states, the metabolic effects of N-TSP1 appear to be context dependent. Our findings suggest that N-TSP1 primarily functions to optimize mitochondrial function and substrate utilization, particularly under metabolic challenges such as HFD-induced obesity, whereas its effects on whole-body energy expenditure and thermogenic outcomes in aged mice or under normal-diet conditions remain to be fully determined. In aged mice, N-TSP1 treatment induced a robust musculoskeletal phenotype characterized by enhanced muscle function, fibre hypertrophy, including hypertrophy of type I fibres, and increased expression of mitochondrial and oxidative metabolism-related markers. These changes were accompanied by molecular signatures consistent with tissue-specific metabolic remodelling in skeletal muscle, ingWAT and BAT. However, they do not establish increased or restored systemic energy expenditure or thermogenesis in aged mice. Rather, these findings support a role for N-TSP1 in promoting adaptive metabolic remodelling in skeletal muscle and adipose tissues. Future studies will be needed to determine whether these molecular adaptations reflect thermogenic heat production, improved functional energy utilization or a combination of both.

Our study identifies N-TSP1 as a distinct proteolytic fragment that contributes to musculoskeletal improvement and tissue-specific metabolic remodelling in response to exercise and cold exposure. Defining its endogenous requirement will be essential for understanding how TSP1 processing contributes to metabolic and musculoskeletal health.

### Study approval

All animal procedures were approved by the Institutional Animal Care and Use Committee (IACUC) at the Korea Research Institute of Bioscience and Biotechnology (KRIBB).

## Supplementary information

Supplementary Information

Dataset1

Dataset2

Dataset3
