# Supplementary material for: N-terminal thrombospondin-1 enhances muscle function and tissue-specific metabolic adaptation in response to exercise and cold exposure
Source: Exp Mol Med. 2026 Sep 8;58(9):3074–87. doi: 10.1038/s12276-026-01830-z (PMC13633276; doi:10.1038/s12276-026-01830-z)
Supplement: Supplementary file 1 [file 12276_2026_1830_MOESM1_ESM.pdf]

**SUPPLEMENTARY INFORMATION**

# N-terminal thrombospondin-1 enhances muscle function and tissue-specific metabolic adaptation in response to exercise and cold exposure

Min Ju Kim[1,2*], Hyun-Jun Jang[3,4*], Jae Sook Kang[1,2*], Sung-Wook Chun[5], Sun Gun Chung[6,7], Shinyeong Ju[8], ByungKuk Min[9], Eunhwan Kim[1], Ju Yeon Kwak[1], Ji-Won Kim[1], Seongwan Kim[1,2], Yeo Jin Shin[1], Kwang-Pyo Lee[1,2,10], Cheolju Lee[8,11], Seung-Min Lee[1], Jung Jae Won[1,2], Eun-Soo Kwon[1,2], Jang Hyun Choi[4,12,#], Ki-Sun Kwon[1,2,10,#], Yong Ryoul Yang[1,2,#]

[#] *Corresponding author*

[*]These authors contributed equally to this work

**Correspondence to:** Jang Hyun Choi, Ki–Sun Kwon, Yong Ryoul Yang

**e–mail:** janghchoi@unist.ac.kr, kwonks@kribb.re.kr, dearyang@kribb.re.kr

**Tel:** +82-42-860-4139

## Supplementary Methods and Materials

### Transcriptomic analyses of skeletal muscle

To identify cold-induced factors secreted from human skeletal muscle, publicly available transcriptomic data (GSE156249) were obtained from the Gene Expression Omnibus database[1]. In this study, only data from healthy participants who underwent skeletal muscle biopsies before and after cold exposure were analyzed, whereas samples from individuals with type 2 diabetes were excluded. As previously published, eight men with type 2 diabetes, aged 59 ± 6 years and with a BMI of 29.9 ± 3.1 kg/m², underwent 10 days of cold exposure (14–15°C) for 2–6 h daily. The raw expression data were normalized using the 'GEOquery version 2.70.0' package in R, and DEGs were identified using the 'Limma version 3.58.1' package. A list of genes encoding secretory proteins was downloaded from The Human Protein Atlas (https://www.proteinatlas.org/), and the expression profiles of these genes were extracted from the cold acclimation dataset.

To identify putative myokines induced by exercise, lists of DEGs from a study of gene expression in skeletal muscle samples before and after training were obtained[2]. Briefly, a human exercise intervention trial was performed as described before[3]. Twenty-six sedentary men (aged 40–65 years, BMI 26 ± 4.0 kg/m²) participated in the trial. Participants underwent 12 weeks of supervised exercise training, including two interval cycling sessions and two strength training sessions per week. Bicycle exercise tests (45 min at 70% of VO2max) were performed before and after the training program. Blood samples and muscle biopsies were collected before, immediately after the acute bicycle tests[2].

### Mouse plasma proteomics

*Preparation of mouse plasma samples*

Four-month-old C57BL/6J (young) male mice underwent a treadmill training program for 5 weeks. The regimen consisted of five 30-min sessions per week at a running speed of 12 m/min. After the training period, plasma samples were collected from 18 sedentary and 18 exercise C57BL/6J mice. Plasma from three mice was pooled for each group, resulting in six pools per group. Abundant plasma proteins (albumin, IgG, and transferrin) were depleted using immunoaffinity chromatography with a MARS-3 column (Agilent). Subsequently, 300 μg of protein from each pool which was blocked its thiols by serial treatment of dithiothreitol and iodoacetamide, was subjected to tryptic digestion, and fractionated into 12 fractions by high pH reversed-phase liquid chromatography (LC). Each fraction was further analyzed by liquid chromatography tandem mass spectrometry (LC-MS/MS).

*LC–MS/MS*

Peptide samples were reconstituted in 0.4% acetic acid and loaded onto a reversed-phase Magic C18aq column (15 cm x 75 μm, 5 μm, 200 Å) housed within an Eksigent NanoLC-Ultra 1D Plus system (Michrom BioResources, Auburn, CA, USA) using a cooled (10°C) autosampler. The flow rate was maintained at 300 nL/min. Before injection, the column was equilibrated with a mobile phase consisting of 95% solvent A (0.1% formic acid in water) and 5% solvent B (0.1% formic acid in acetonitrile). Peptide elution was achieved using a linear gradient, ramping from 5% to 40% solvent B over 120 min. This was followed by an organic wash step and column re-equilibration with solvent A, all at a flow rate of 300 nL/min. The total run time was 170 min. The LC system was interfaced with a Q Exactive quadrupole mass spectrometer (Thermo Fisher Scientific, Bremen, Germany) operating in a data-dependent acquisition (DDA) mode. High-resolution (70,000) full-scan mass spectra (m/z 300–2000)

were acquired. The source ionization parameters were optimized as follows: spray voltage, 1.9 kV; capillary temperature, 275°C; and s-lens level, 44.0. The full width at half maximum of the MS peaks was maintained below 30 s. The 12 most abundant ions with a charge state of ≥ 2 from the MS1 scan were selected for fragmentation at a dynamic exclusion duration of 90 s using the following settings: resolution, 17,500; isolation window, 2.0 m/z; normalized collision energy, 27%; ion threshold, 4.00E+03 counts; and precursor ion selection favoring matched peptides. The injection volumes were adjusted to ensure consistent loading of approximately 1 μg of tryptic peptides.

*Protein Identification and Quantitation*

Protein identification and quantitation from the LC-MS/MS data were performed by using MaxQuant (v1.5.8.3) software with UniProt mouse protein database (v2016.07) to which appended contaminant protein sequences of human keratins and common laboratory proteases. The parameters for the database search are: protein FDR, 0.01; minimal unique peptides, 1; minimal razor peptides, 1; match between runs, True; minimal matching time window, 0.7 min; alignment time window, 20 min; decoy mode, revert; fixed modification, carbamidomethyl of Cys; variable modifications, oxidation of Met, acetyl of protein N-term. For other undescribed parameters, we used program default values.

*Thrombospondin-1 (TSP1) protein domain-level quantitation*

Peptide quantitation result of MaxQuant output was used to elucidate TSP1 domain-level fold change. TSP1 (UniProt accession of P35441) peptides start from 1–259 position was set as N-terminal domain (N-TSP1), 260 to 690 position as middle domain (M-TSP1) and after 691 position as C-terminal domain (C-TSP1). Fold change was calculated by summing the

intensities of domain-specific peptides each individual mouse, and compared the summed intensity between pre- and post-exercise data of same mouse. Significance was calculated using a two-sided Student's *t*-test. The fold changes were visualized using GraphPad Prism version 10 (GraphPad Software, San Diego, CA, USA).

**Acute treadmill exercise for mouse**

To acclimate the mice to the treadmill before initiating the acute training protocol, a daily habituation period was implemented for five consecutive days. Each habituation session consisted of a 10-min run at a speed of 10 m/min. During the subsequent formal training sessions, the mice were placed on the treadmill belt, and the running speed was progressively increased by 2 m/min every 2 min, culminating in a final challenging speed of 24 m/min with a 10% incline. This exercise bout was maintained for a total duration of 1 h. This training regimen was sustained for 1 week. Blood was collected from mice immediately after 30, 60, and 90 min of exercise without any rest period on the final day of training. Blood from the 0 min group was also collected from sedentary control mice. Tissues were collected only after 30 min of acute treadmill exercise.

**Mouse cold exposure**

For cold exposure studies, mice were transferred from 23–25°C to 4°C for 6 h. Mice were singly housed without nesting material and had ad libitum access to food and water. Blood and tissue samples were harvested immediately upon removal from the cold room.

**Administration of N-TSP1 in vivo**

Recombinant mouse N-TSP1 protein, a peptide spanning amino acids 19–259 (Y-biologics), was used for all experiments. Briefly, diet-induced obese mice were fed a high-fat diet (HFD) for two weeks prior to protein injections. Subsequently, obese mice received daily intraperitoneal (i.p.) injections of N-TSP1 (0.5 mg/kg/day) or PBS (vehicle) for eight weeks (n = 7–8 mice per group). 23-month-old mice were administered daily i.p. injections of N-TSP1 (0.5 mg/kg/day) or PBS (vehicle) for 15 days (n = 6–8 mice per group). 12-week-old mice were supplemented with N-TSP1 (0.5 mg/kg/day) or PBS (vehicle) for 28 days (n = 8 per group).

**Cell culture and adipogenic differentiation**

The C2C12 mouse myoblast cell line (CRL-1772, ATCC, female) was cultured in a humidified incubator at 37°C with 5% $CO_2$. Cells were grown in Dulbecco's Modified Eagle's Medium (DMEM, LM001-05, Welgene) supplemented with 10% fetal bovine serum (FBS, 16000044, Gibco), 100 U/mL penicillin, and 100 μg/mL streptomycin (LS202-02, Welgene). Differentiation into myotubes was induced by switching to DMEM containing 2% FBS, penicillin, and streptomycin for 3 days. To mimic exercise effects, differentiated C2C12 myotubes were treated with 1 mM AICAR (A9978, Sigma), 1 μM forskolin (F6886, Sigma), and 1 μM ionomycin (I0634, Sigma) for 24 h. To verify the specificity of the TSP1 antibodies, differentiated C2C12 myotubes were transfected with siRNA targeting Thbs1 (21825, Bioneer). Following transfection, myotubes were maintained in serum-free medium for 24 h, after which both cell lysates and conditioned media were harvested. The influence of N-TSP1 on myogenesis was evaluated by differentiating C2C12 cells into myotubes in 2% FBS DMEM containing varying concentrations of N-TSP1 (100, 200, and 500 ng/mL) for 3 days. Human skeletal muscle myoblasts (CC2580, Lonza) isolated from a 19-year-old donor were expanded in Skeletal Muscle Cell Growth Medium-2 (CC-3246, Lonza) supplemented with a supplement

pack (CC-3244, Lonza) containing GA-1000, human epidermal growth factor (hEGF), dexamethasone, L-glutamine, and 10% FBS (16000044, Gibco). Differentiation was achieved using DMEM/F12 (11320-033, Gibco) with antibiotics and 2% horse serum (26050088, Gibco).

An immortalized brown preadipocyte cell line was generously provided by Dr. Shingo Kajimura (UCSF, San Francisco, CA, USA). Cells were cultured in DMEM/F-12 with GlutaMAX supplement (10565018, Gibco) containing 10% FBS (Gibco) and 1% antibiotics at 37°C in a humidified atmosphere with 5% $CO_2$. To induce brown adipocyte differentiation, cells were cultured to confluence (day 0) in a maintenance medium containing 10% FBS and 1% antibiotics. Subsequently, cells were differentiated using a differentiation medium (DMEM/F-12 with GlutaMAX supplement containing 10% FBS) supplemented with 0.5 mM isobutylmethylxanthine (IBMX, I5879, Sigma), 0.5 μM dexamethasone (D2915, Sigma), 20 nM insulin (I9278, Sigma), 1 nM 3,3',5-Triiodo-L-thyronine (T3, T2877, Sigma), and 125 μM indomethacin (I7378, Sigma) for 2 days. Following this 2-day induction period (day 2), cells were further differentiated and matured for an additional 2 days in a growth medium composed of DMEM, 10% FBS, 20 nM insulin, and 1 nM T3. Mature brown adipocytes were treated with or without 1 μM isoproterenol (ISO, I6504, Sigma), a non-selective β-adrenergic agonist, for 24 h.

**Immunocytochemistry**

C2C12 myoblasts were seeded in 24-well plates and differentiated into myotubes. Following differentiation, myotubes underwent a gentle PBS wash, followed by fixation for 15 min at room temperature with 4% paraformaldehyde. Three subsequent washes with PBS ensured complete removal of the fixative. Permeabilization was achieved by exposure to 0.25% Triton X-100 in PBS for 10 min. To minimize background staining, myotubes were blocked for 30

min with 1% BSA in PBS containing 0.1% Tween-20. This was followed by overnight incubation at 4°C with an anti-MYH primary antibody (SC-376157, Santa Cruz) diluted in blocking buffer. After primary antibody incubation, myotubes were incubated for 1 h at room temperature with an Alexa Fluor® 488-conjugated secondary antibody (A-21121, Thermo Fisher) diluted in 1% BSA in PBS. Nuclei were visualized by staining for 1 min in the dark with DAPI-containing Vectashield mounting medium (H-1200, VECTOR Laboratories). Images were captured under a Nikon Eclipse Ti-U inverted microscope equipped with a Nikon DS-Ri2 camera and NIS-Elements software. Myotube diameter was quantified by measuring the short axis using ImageJ software.

**Measurement of the oxygen consumption rate (OCR)**

Mitochondrial respiration in C2C12 myotubes was assessed using the Seahorse XF Cell Mito Stress Test Kit (103015-100, Agilent Technologies) on a Seahorse XFe96 Analyzer (Agilent Technologies). C2C12 cells were seeded in Seahorse XFe96/XF Pro Cell Culture Microplates (103794-100, Agilent Technologies) and differentiated in DMEM supplemented with 2% FBS under confluent conditions. After one day of differentiation, myotubes were treated with 1 μg/mL each of F-TSP1, N-TSP1, M-TSP1, or C-TSP1 in DMEM with 2% FBS, with the medium was changed daily for three consecutive days. Following the treatment period, cells were incubated for 30 min in Seahorse XF DMEM assay medium (pH 7.4) before initiating the assay. The Mito Stress Test was conducted according to the manufacturer's protocol, involving sequential injections of oligomycin (1.5 μM) to inhibit ATP synthase, FCCP (1 μM) to uncouple mitochondrial membrane potential and assess maximal respiration, and a combination of rotenone and antimycin A (0.5 μM each) to inhibit Complex I and Complex III, respectively, thereby measuring non–mitochondrial respiration. After completing the oxygen consumption

rate (OCR) assay, the assay medium was carefully removed for protein normalization. Wells were rinsed with phosphate-buffered saline (PBS) to remove residual media. RIPA buffer (CBR002, LPS solution) (100 µL) was added to each well, and the plate was incubated on ice for 10 min to ensure complete cell lysis. To enhance lysis efficiency, lysates were subjected to gentle pipetting. Protein content was quantified using the bicinchoninic acid (BCA) assay (23228, Thermo Scientific, Pierce™ BCA Protein Assay Reagent A; 1859078, Pierce™ BCA Protein Assay Reagent B). A standard curve was prepared using bovine serum albumin (BSA) standards. Lysates and standards were plated in duplicate in a 96-well plate, and absorbance was measured at 562 nm using a microplate reader (Bio-Tek, Synergy HT Multi-detection microplate reader). OCR values were normalized to the protein concentration (e.g., pmol/min x µg protein).

**Cell proliferation assay**

Cell proliferation was assessed in real time using the Incucyte Live-Cell Analysis System (Sartorius). Cells were seeded at 1 x $10^3$ cells per well into a 96-well plate (30096, SPL) at 20% confluence to maintain logarithmic growth throughout the experiment. After cell attachment, treatments with N-TSP1 at concentrations of 1, 2, or 4 µg/mL were applied. The plate was transferred to the Incucyte Live-Cell Imaging System, and phase–contrast images were captured every 12 h over a 2-day period. Cell confluence (%) was quantified using the Incucyte's integrated image analysis software, which calculated the cell-covered area relative to the total well area. Growth curves were generated by plotting confluence over time, and proliferation rates were expressed relative to Day 0 measurements. All experiments were performed in triplicate to ensure reproducibility and statistical reliability.

**Electrical pulse stimulation (EPS)**

Following a 3-day differentiation period, C2C12 myotubes cultured in six-well plates were subjected to an electrical stimulation protocol using a C-PACE EP culture pacer (IonOptix, Dublin, Ireland) at 37°C. The stimulation protocol consisted of 1-ms pulses delivered at a frequency of 1 Hz for a 2-ms duration, with an applied voltage of 11.5 V. This electrical stimulation regimen was administered for 24 h, followed by a 1-hour incubation period for recovery.

**Fusion index**

C2C12 myoblasts were differentiated into myotubes in the presence of varying concentrations of N–TSP1 (0, 100, 200, and 500 ng/mL) or vehicle control (PBS). Immunocytochemistry was performed using an anti-MYH primary antibody and DAPI staining. The myotube fusion index was calculated by dividing the number of nuclei within myotubes by the total number of nuclei within the field of view and multiplying the result by 100. Fiber diameter was determined by averaging 20 different measurements along the myotube in a field of view at 200 x magnification.

**Western blotting**

Cell and tissue lysates were prepared using radioImmunoprecipitation assay (RIPA) buffer supplemented with protease and phosphatase inhibitors (0.1 mM $Na_3VO_4$, 1 mM NaF, 1 mM AEBSF, and 5 mg/mL aprotinin). Total protein concentration was measured using bicinchoninic acid (BCA) and Bradford assays. Samples were then resolved by SDS-PAGE and electrotransferred to nitrocellulose membranes. To minimize nonspecific binding,

membranes were incubated for 1 hour at room temperature with either 5% non-fat dry milk or 2% bovine serum albumin (BSA) in Tris-buffered saline. Following overnight incubation at 4°C with primary antibodies diluted 1:1000, membranes were washed and probed for 1 h at room temperature with horseradish peroxidase (HRP)-conjugated secondary antibodies. Protein bands were visualized using a chemiluminescence imaging system. Primary antibodies are provided in Supporting Information (Supplementary Table 1).

**RNA isolation and quantitative RT-PCR (qPCR)**

RNA was isolated from muscle tissues, other tissues, C2C12 myotubes, and brown adipocytes using the RiboEx reagent (301-001, GeneAll Biotechnology, South Korea) and an RNA isolation kit (305-101, GeneAll). From isolated RNA, cDNA was synthesized using iScript™ cDNA Synthesis Kit (1708897, Bio-Rad). Quantitative real–time PCR was performed using a StepOnePlus™ Real-Time PCR System (Applied Biosystems). Each 20 µL reaction contained cDNA, gene-specific primers, and SYBR Green Master Mix (Applied Biosystems). All gene expression data were normalized to *RPLP0* (36B4). Primer sequences are provided in Supporting Information (Supplementary Table 2).

**Isolation and adipocyte differentiation of SVFs**

Dissected adipose tissues were processed for stromal vascular fraction (SVF) isolation as previously described in our paper[4], with modifications. Adipose tissues were finely minced with scissors in 1 mL of digestion media per gram of tissue. The digestion media consisted of phosphate-buffered saline (PBS) containing 1% bovine serum albumin (BSA), 3 mg/mL collagenase type 1 (SCR103, Sigma-Aldrich), 5 mg/mL dispase II (D4693, Sigma-Aldrich), and 10 mM calcium chloride ($CaCl_2$). The mixture was incubated in a shaking water bath at

37°C for 40 min. The digested tissue was then mixed with an equal volume of Dulbecco's Modified Eagle Medium (DMEM)/F-12 supplemented with 10% fetal bovine serum (FBS) and centrifuged at 700 x g for 10 min. The pelleted SVFs were resuspended in DMEM/F-12 and filtered through a 100-μm nylon mesh strainer (Falcon). The SVFs were pelleted again by centrifugation as above and resuspended in DMEM/F-12 supplemented with 10% FBS. The SVFs were then transferred to culture plates for further experiments.

The isolated SVFs were maintained in DMEM/F-12 supplemented with 10% FBS, 100 U/mL penicillin, and 100 μg/mL streptomycin. To differentiate into brown-like adipocytes, confluent SVFs were cultured with an induction cocktail consisting of 200 nM insulin (I0516, Sigma-Aldrich), 0.5 μM dexamethasone (D4902, Sigma-Aldrich), 500 μM 3-isobutyl-1-methylxanthine (I5879, Sigma-Aldrich), 125 μM indomethacin (I7378, Sigma-Aldrich), and 1 nM T3 (T6397, Sigma-Aldrich) for 2 days. After 2 days, the cells were switched to a maintenance medium composed of DMEM/F-12 supplemented with 10% FBS, 100 U/mL penicillin, 100 μg/mL streptomycin, 200 nM insulin, and 1 nM T3. The cells were differentiated into brown-like adipocytes for an additional 6 days, with the medium being changed every 2 days. Rosiglitazone 1 μM (R2408, Sigma-Aldrich), for 24-h treatment served as a positive control for induction of thermogenesis.

**Puromycin incorporation assay**

Myotube protein synthesis was assessed using a puromycin incorporation assay. Puromycin (Sigma P8833) was added to the cell culture medium at a final concentration of 1 μM, 30 min prior to protein collection. The extent of puromycin incorporation into newly synthesized proteins was determined by Western blotting.

**Measurement of rectal temperature**

A flexible probe (Testo 925, DG-08GS) was inserted to measure rectal temperature. Throughout the experiment, mice were housed at room temperature (25℃) and 18°C. The inserted probe was held steady for approximately 10 seconds. The rectal temperature displayed on the thermometer was recorded.

**Food intake, energy expenditure, and body composition**

Measurement of cumulative food intake, locomotion, and $VO_2$ consumption was performed using a Comprehensive Lab Animal Monitoring System at room temperature (20–22°C) (Oxymax; Columbus Instruments). Singly housed mice were acclimated in metabolic chambers for at least 24 h before the start of experiments to minimize stress. Fat and lean mass were measured by EchoMRI.

**Histological analysis**

Liver tissues were fixed in 4% paraformaldehyde and then embedded in paraffin. All tissues were cut into 6-μm sections and stained with hematoxylin and eosin (H&E) and Oil Red O (ORO) solution using standard methods. The relative area occupied by hepatic vacuoles in H&E and ORO-stained samples was analyzed by Image J software.

**Intracellular lipid accumulation**

The HepG2 cells (HB-8065, ATCC, male) were cultured in a humidified incubator at 37°C, 5% $CO_2$. Cells were grown in Dulbecco's Modified Eagle Medium (DMEM, LM001-05, Welgene) supplemented with 10% fetal bovine serum (FBS, 16000044, Gibco), 100 U/mL penicillin, and 100 μg/mL streptomycin (LS202-02, Welgene). For intracellular lipid accumulation, HepG2

cells were cultured with 1 mM sodium oleic acid (OA) or 500 ng/mL N-TSP for 24 h. The lipid droplets were stained with Oil Red O (ORO) and analyzed by Image J software.

**Immunohistology**

Muscle samples at resting length were embedded in an optimal cutting temperature (OCT) medium (4583**,** Tissue-Tek) and frozen rapidly in liquid nitrogen before storage at -80°C. Following freezing, 7 μm-thick sections were obtained from the mid-belly region using a cryostat (CM1950, Leica) maintained at -20°C. The sections were carefully mounted on microscope slides (5116-20F, MUTO, Japan) and stored at -80°C. For immunofluorescence staining, consecutive sections were air-dried for 30 min and then washed three times with PBS. The sections then underwent a combined blocking and permeabilization step by incubation in PBS containing 0.4% Triton X-100 for 30 min. Subsequently, sections were incubated with a cocktail of primary antibodies specific for laminin (L9393, Sigma-Aldrich), MYH isoforms MYH7 (BA-F8, DSHB), MYH2 (BF-32, DSHB), and MYH4 (BF-F3, DSHB) in PBS containing 10% goat serum (ab7481, Abcam) for 2 h at room temperature. This was followed by incubation with Alexa Fluor 555-conjugated secondary antibodies (A32732 and A28180, Invitrogen) diluted in PBS containing 10% goat serum. After three final washes in PBS, sections were mounted with a DAPI-containing Vectashield mounting medium (H-1200, VECTOR Laboratories). Images were acquired under a Nikon Eclipse Ti-U inverted microscope equipped with a Nikon DS-Ri2 camera and NIS-Elements software.

**Glucose and insulin tolerance tests**

Metabolic assays were performed with standardized fasting protocols tailored to each test. For Glucose tolerance tests (GTT), mice were fasted for 12 h starting at 21:00 (during the dark

phase), with free access to water, before i.p. administration of D-glucose (Sigma, 2 g/kg body weight) at 09:00 the following morning. For Insulin tolerance tests (ITT), mice underwent a 6-h fast starting at 08:00 (during the light phase), with free access to water, before insulin (0.75 U/kg body weight) i.p. injection at 14:00. In both experiments, cage bedding was replaced at the time of food removal. Blood glucose levels were measured at baseline, 15, 30, 60, 90, and 120 min using an Accu-Chek Performa Blood Glucose Meter. Mice were maintained under a 12-h light/dark cycle (lights off at 16:00 and lights on at 04:00). This difference in fasting duration and circadian timing may account for the higher baseline glycemia observed in the ITT cohorts compared to the GTT cohorts.

**Biochemical analysis (Plasma analysis)**

Blood samples were centrifuged and plasma was collected for analysis. All parameters were determined by an AU480 Chemistry Analyzer (Beckman Coulter).

**Statistical analysis**

All data are presented as means ± standard error of the mean (SEM). Normality was assessed using the Shapiro-Wilk test prior to statistical analyses. For normally distributed data, statistical comparisons were performed using two-tailed paired or unpaired Student's *t*-test and one-way ANOVA with Dunnett's post–hoc test. For non-normally distributed data, Mann-Whitney U test was employed in place of the Student's *t*-test. Additional statistical analyses included repeated-measures ANOVA, two-way ANOVA and two-way repeated-measures ANOVA with Tukey's post-hoc tests, Log-rank (Mantel-Cox) test for survival analysis, and Pearson's correlation coefficient. Statistically significant differences are defined as $*P < 0.05$, $**P < 0.01$, $***P < 0.005$, $****P < 0.001$. Data analysis was performed using GraphPad Prism version 10

(GraphPad Software, San Diego, CA, USA).

## References


1 Nascimento, E. B. M. *et al.* Comparative transcriptome analysis of human skeletal muscle in response to cold acclimation and exercise training in human volunteers. *BMC Med Genomics* **13**, 124 (2020). https://doi.org/10.1186/s12920-020-00784-z

2 Pourteymour, S. *et al.* Global mRNA sequencing of human skeletal muscle: Search for novel exercise-regulated myokines. *Mol Metab* **6**, 352-365 (2017). https://doi.org/10.1016/j.molmet.2017.01.007

3 Langleite, T. M. *et al.* Insulin sensitivity, body composition and adipose depots following 12 w combined endurance and strength training in dysglycemic and normoglycemic sedentary men. *Arch Physiol Biochem* **122**, 167-179 (2016). https://doi.org/10.1080/13813455.2016.1202985

4 Jang, H. J. *et al.* Glucosylceramide synthase regulates adipo-osteogenic differentiation through synergistic activation of PPARgamma with GlcCer. *FASEB J* **34**, 1270-1287 (2020). https://doi.org/10.1096/fj.201901437R

**Supplementary Table 1. List of Antibodies Used for Western Blot Analysis**

| Primary antibody | company | Cat# |
|---|---|---|
| AKT | SCBT | SC1618 |
| mTOR | CST | 2983S |
| p70S6K | CST | 9202S |
| FOXO3a | CST | 12829S |
| PRDM16 | Thermo | PAS-20872 |
| UCP1 | Abcam | Ab10983 |
| PPARγ | CST | 2435S |
| p38 | SCBT | SC-535 |
| CREB | CST | 9197S |
| Anti-C-terminal -TSP1 | Abcam | ab1823 |
| Anti-N-terminal-TSP1 | USCN | PAA611mu01 |
| OXPHOS cocktail | Invitrogen | 45-8099 |
| Puromycin | DSHB | PMY-2A4 |
| FAK | BD | 610087 |
| Src | CST | 2108T |
| Actin | SCBT | SC-1616 |
| α-Tubulin | CST | 2144 |
| GAPDH | Sigma | G9545 |
| p-AKT (S473) | CST | 9271S |
| p-mTOR (S2448) | CST | 5536S |
| p-p70S6K | CST | 9206S |
| p-FOXO3a | CST | 13129S |
| p-PPARγ (S273) | Bioss | BS-4888R |
| p-p38 | CST | 9211S |
| p-CREB | CST | 9198S |
| p-FAK | CST | 3284T |
| p-Src | abcam | ab4816-50 |

**Supplementary Table 2. List of All Primer Sequences Used for qRT-PCR**

| Gene | Forward (5'→3') | Reverse (5'→3') |
|---|---|---|
| *Rplp0* | AGA TTC GGG ATA TGC TGT TGG | AAA GCC TGG AAG AAG GAG GTC |
| *Tsp1* | GGG GAG ATA ACG GTG TGT TTG | CGG GGA TCA GGT TGG CAT T |
| *Tsp2* | TCG GAC CTC AAG TAT GAG TGC | TCT AAG AAG GGG TGT TTG CAG |
| *Tsp3* | TCG CCA AGA CAA CAC ACG | CAT GGA CTT TGC CAT CTT CC |
| *Tsp4* | AGA CGC CTG TGA TGA CGA C | TGG GAC AGT TGT CCA AAA TG |
| *Tsp5* | CTG CTC CGA GAC AGC TAC G | GGG TGA GCA TTG CAC TCA |
| *Ucp1* | GTG AAC CCG ACA ACT TCC GAA | TGA AAC TCC GGC TGA GAA GAT |
| *Elovl3* | TTC TCA CGC GGG TTA AAA ATG G | TCT CGA AGT CAT AGG GTT GCA T |
| *Ascl1* | CGGAACTGATGCGCTGCAAACG | GGCAAAACCCAGGTTGACCAAC |
| *Cpt1b* | CCC ATG TGC TCC TAC CAG AT | CCT TGA AGA AGC GAC CTT TG |
| *Prdm16* | ATCCACAGCACGGTGAAGCCAT | ACATCTGCCCACAGTCCTTGCA |
| *Acox1* | TAA CTT CCT CAC TCG AAG CCA | AGT TCC ATG ACC CAT CTC TGT C |
| *Atp5b* | TCCTGCCAGAGACTATGCG | GATGACTGCCACGATTCGC |
| *Atp5o* | CTATGCAACCGCCCTGTACTCT | CACTTTGACGGTGCGCTTGATG |
| *Cidea* | GGTGGACACAGAGGAGTTCTTTC | CGAAGGTGACTCTGGCTATTCC |
| *Acc1* | GTTCTGTTGGACAACGCCTTCAC | GGAGTCACAGAAGCAGCCCATT |
| *Pgc1a* | GGACATGTGCAGCCAGAGCTC | CACTTCAATCCACCCAGAAAGCT |
| *Cox5b* | TTC AAG GTT ACT TCG CGG AGT | CGG GAC TAG ATT AGG GTC TTC C |
| *Scd1* | TAG CCT GTA AAA GAT TTC TGC AAA CC | CCG GAG ACC CTT AGA TCG A |
| *Fas* | CCT GGA TAG CAT TCC GAA CCT | GCA CAT CTC GAA GGC TAC ACA |
| *Glut4* | CTT GGC TCC CTT CAG TTT GG | CTA CCC AGC CAC GTT GCA TT |
| *Tfam* | GGA ATG TGG AGC GTG CTA AAA | ACA AGA CTG ATA GAC GAG GGG |
| *Pparα* | CCT GAA CAT CGA GTG TCG AAT AT | GGT TCT TCT TCT GAA TCT TGC AGC T |
| *Fasn* | TCC TGG GAG GAA TGT AAA CAG C | CAC AAA TTC ATT CAC TGC AGC C |
| *Gpam* | ACA GTT GGC ACA ATA GAC GTT T | CCT TCC ATT TCA GTG TTG CAG A |
| *CD36* | TCC AGC CAA TGC CTT TGC | TGG AGA TTA CTT TTC AGT GCA GAA |
| *Fabp2* | AAA GGA AAC CTC ATT GCC ACC A | AAT GTC GCC CAA TGT CAT GGT A |
| *Mttp* | ATA CAA GCT CAC GTA CTC CAC T | TCC ACA GTA ACA CAA CGT CCA |
| *Nr1h3* | GCT CTG CTC ATT GCC ATC AG | TGT TGC AGC CTC TCT ACT TGG A |
| *Acaca1* | GCC ATT GGT ATT GGG GCT TAC C | CCC GAC CAA GGA CTT TGT TG |
| *Srebf1* | GGA GCC ATG GAT TGC ACA TT | CCT GTC TCA CCC CCA GCA TA |
| *Mlxiip1* | CTG GGG ACC TAA ACA GGA GC | GAA GCC ACC CTA TAG CTC CC |
| *Slc2a2* | TCA GAA GAC AAG ATC ACC GGA | GCT GGT GTG ACT GTA AGT GGG |

| | | |
|---|---|---|
| *Pnpla2* | CAA CGC CAC TCA CAT CTA CGG | GGA CAC CTC AAT AAT GTT GGC AC |
| *Lipa* | TGT TCG TTT TCA CCA TTG GGA | CGC ATG ATT ATC TCG GTC ACA |
| *Atg3* | ACA CGG TGA AGG GAA AGG C | TGG TGG ACT AAG TGA TCT CCA G |
| *Atg5* | AGC CAG GTG ATG ATT CAC GG | GGC TGG GGG ACA ATG CTA A |
| *Sqstm1* | AGG ATG GGG ACT TGG TTG C | TCA CAG ATC ACA TTG GGG TGC |
| *Map1lc3b* | TTA TAG AGC GAT ACA AGG GGG AG | CGC CGT CTG ATT ATC TTG ATG AG |
| *Lipe* | TCA ACC GAC CAG CAG TGC TC | CTC TGG GTC TAT GGC GAA TC |

## Supplementary Figures and Figure Legends

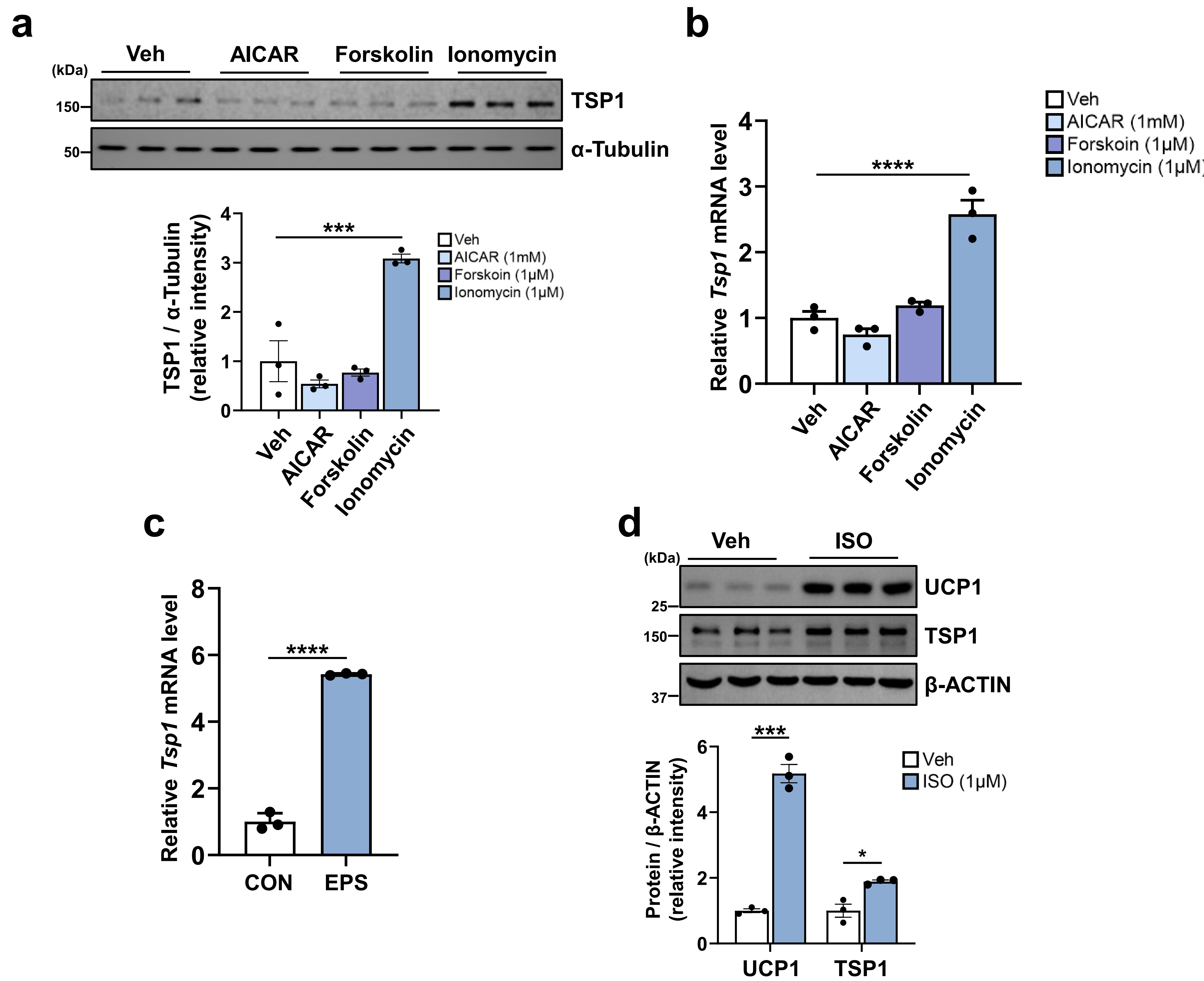


**Supplementary Fig. 1. Expression of TSP1 in myotubes treated with AICAR, forskolin, and ionomycin, and in differentiated brown adipocytes treated with isoproterenol (ISO).**

(a) TSP1 expression in C2C12 myotubes treated with 1 mM AICAR, 1 μM forskolin, and 1 μM ionomycin for 24 h after 3 days of differentiation (upper). Relative densitometric quantification of F-TSP1 protein expression level (n = 3 per group) (lower). (b) The mRNA expression level of *Tsp1* in C2C12 myotubes under conditions identical to (a) (n = 3 per group). (c) The mRNA expression of *Tsp1* in C2C12 myotubes stimulated by electrical pulse stimulation (EPS) (n = 3 per group). (d) Expression levels of UCP1 and TSP1 in differentiated brown adipocytes. Brown adipocytes were treated with ISO (1 μM) for 24 h after 4 days of differentiation (upper). Relative densitometric quantification of Western blot analysis. β-actin was used as a loading control (n = 3 per group) (lower). Data are presented as mean ± SEM. *P*-values were calculated using one-way ANOVA with Dunnett's multiple comparison post hoc test (a and b) and two-tailed unpaired Student's *t*-test (c and d) following Shapiro–Wilk normality test. (*$P < 0.05$, ***$P < 0.001$, ****$P < 0.0001$).

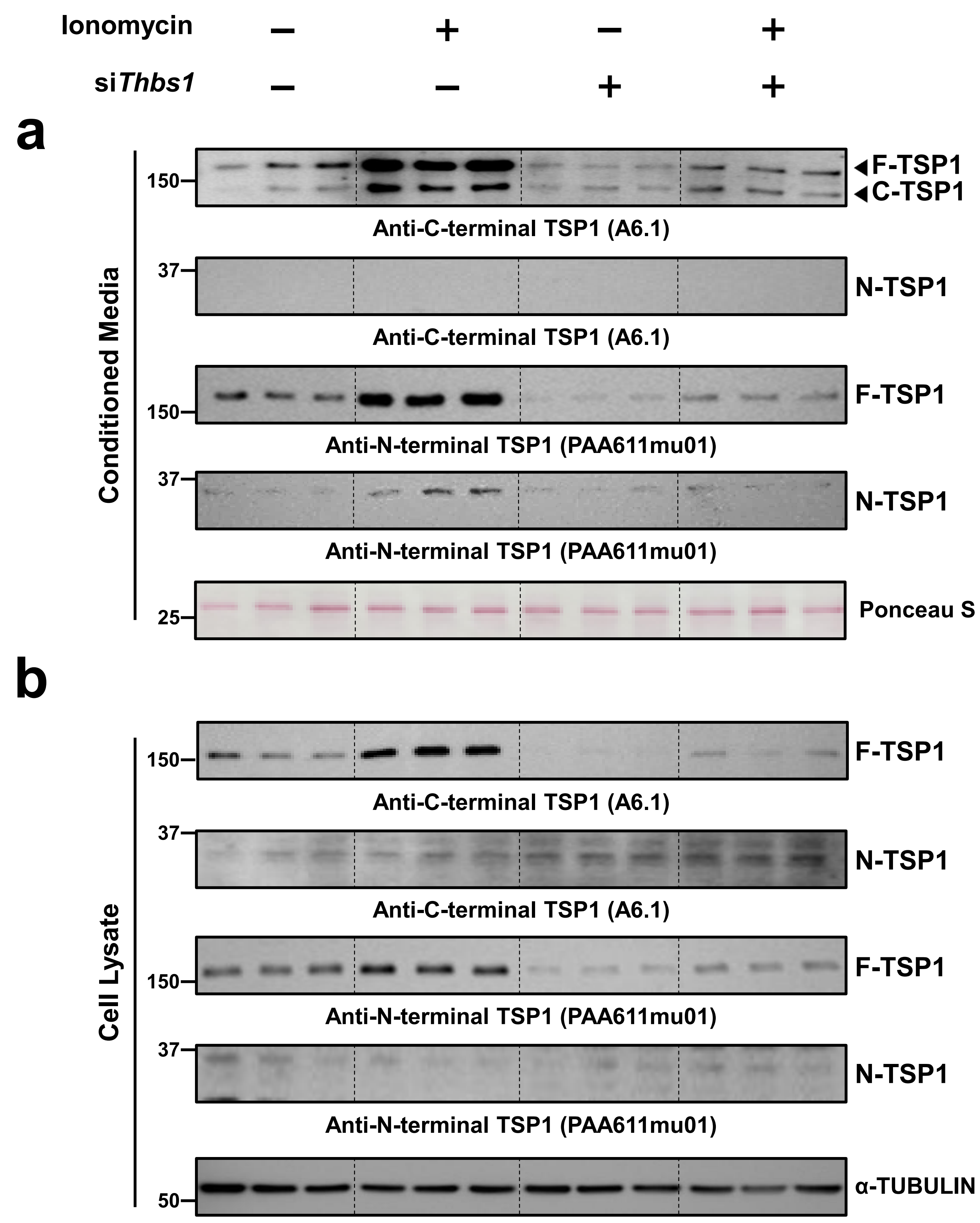


**Supplementary Fig. 2.** Validation of anti-TSP1 antibodies in cell lysates and conditioned media from ionomycin- and si*Thbs1*-treated C2C12 myotubes.

C2C12 myotubes were treated with ionomycin in the presence or absence of si*Thbs1*, and TSP1-related fragments were analyzed by immunoblotting using antibodies recognizing N-TSP1 and C-TSP1.

(a) Immunoblotting of serum-free conditioned media of each well was collected after 24 h of treatment (n = 3 per group). (b) Immunoblotting of cell lysates from C2C12 myotubes treated with ionomycin (1 μM) and si*Thbs1* (30 pmol per well) were treated for 24 h. α-tubulin was used as a loading control (n = 3 per group). si*Thbs1*, siRNA targeting *Thbs1*, the gene encoding mouse TSP1.

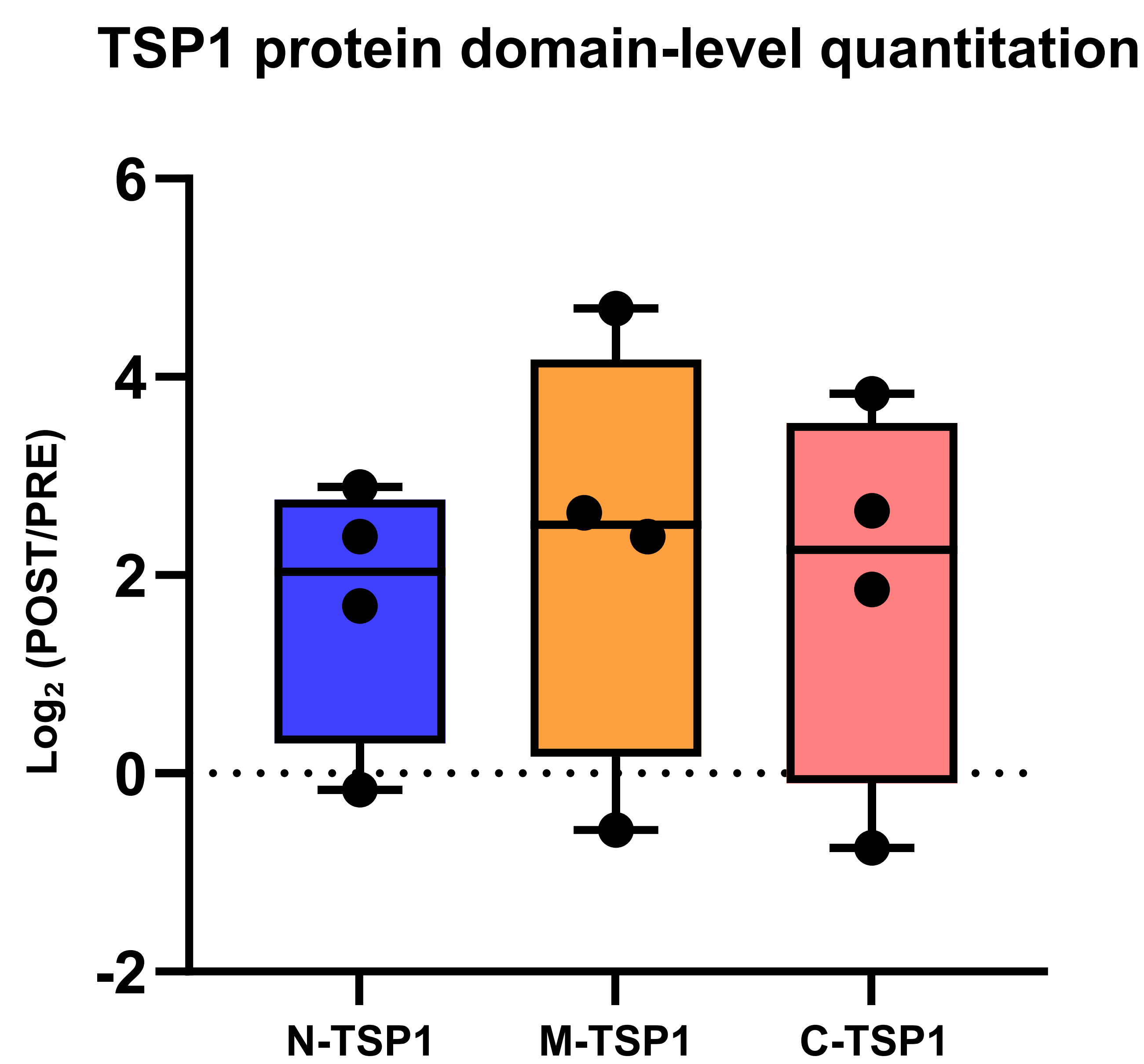


**Supplementary Fig. 3. Domain-specific fold changes in TSP1 abundance between pre- and post-exercise mouse plasma model.**

Boxplots show domain-level fold changes in TSP1 peptide abundance between pre- and post-exercise mouse plasma samples. Repeated-measures ANOVA was used to assess the significance.

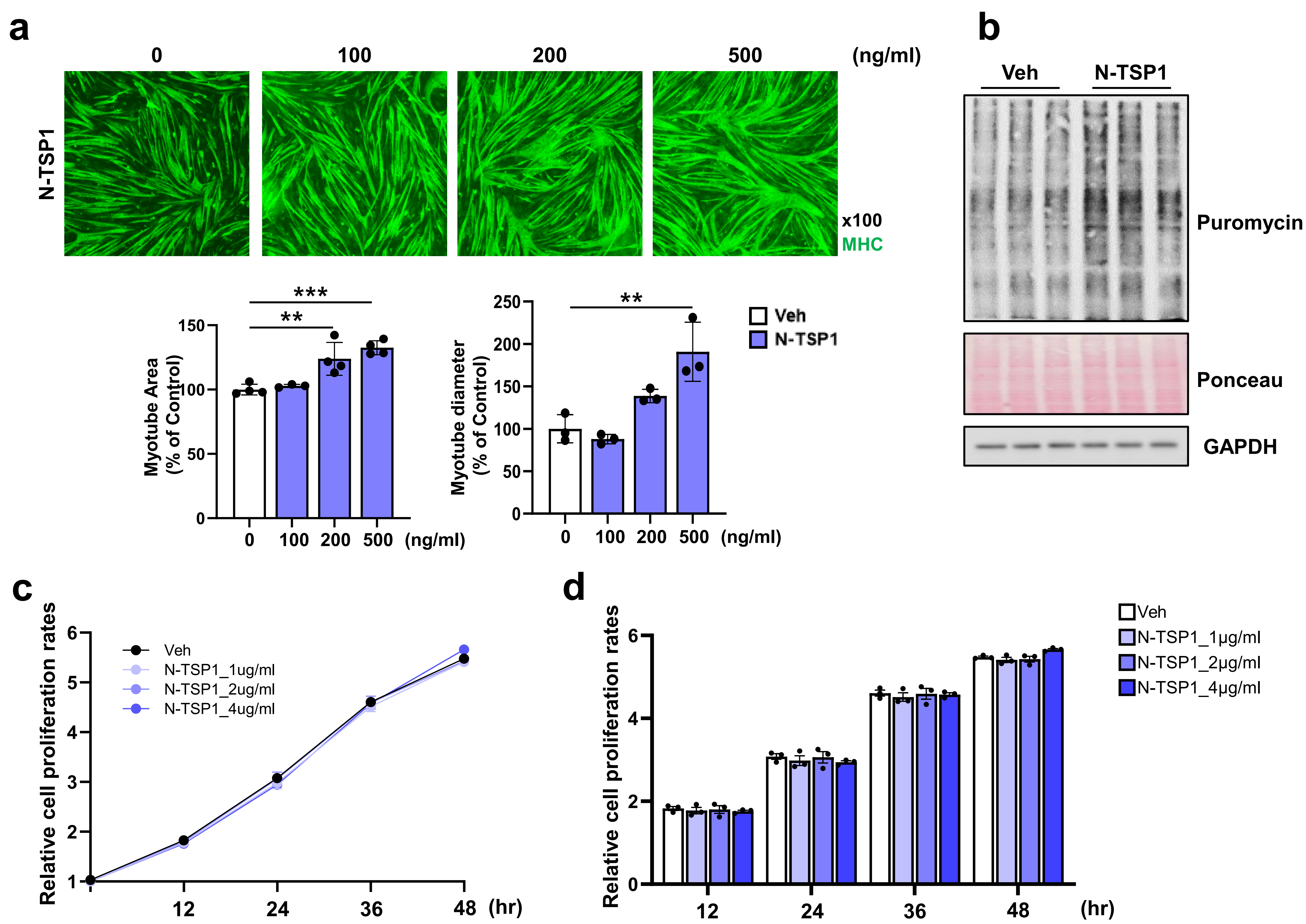


**Supplementary Fig. 4. N-TSP1 promotes hypertrophy and protein synthesis in C2C12 myotubes without affecting cell proliferation.**

(a) Representative images of myosin-stained C2C12 myotubes differentiated for 3 days in a medium containing different doses of N-TSP1 (0, 100, 200, and 500 ng/mL) (upper), followed by quantification of myotube area and diameter (n = 3 per group) (lower). (b) A puromycin incorporation assay was performed in C2C12 myotubes treated with N-TSP1 500 ng/ml (n = 3 per group). (c) Relative cell proliferation was measured every 12 h for 2 days after C2C12 myoblast seeding. Relative cell proliferation rates were normalized to the 0 h measurement. (d) Relative cell proliferation rates in panel c are depicted in the bar graphs (n = 3 per group). Data are presented as mean ± SEM. *P*-values were calculated using one-way ANOVA with Dunnett's multiple comparison post hoc test following Shapiro–Wilk normality test (a, c, and d) (***P* < 0.01, ****P* < 0.001).

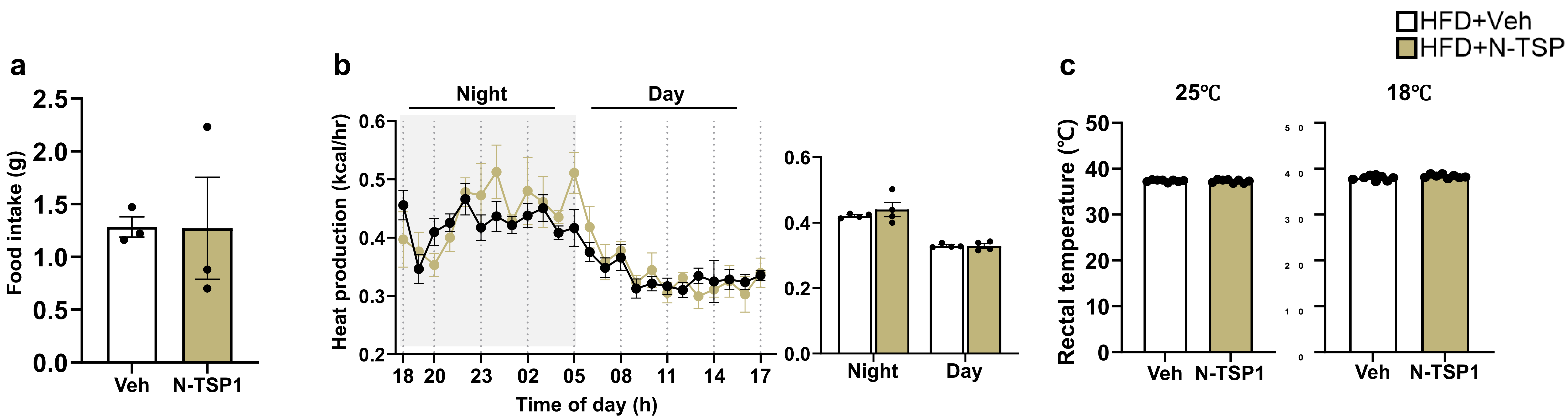


**Supplementary Fig. 5. Changes in food intake, heat production, and rectal temperature were assessed in HFD-fed mice injected with N-TSP1 or vehicle.**

Mice were placed in CLAMS cages for at least 2 days. (a) Food intake measurement and (b) Heat production of HFD groups treated with vehicle or N-TSP1 (0.5 mg/kg/day) over 24 h after 8 weeks of supplementation. (c) Rectal temperature of HFD mice treated with vehicle or N-TSP1 (0.5 mg/kg/day) at 25°C and 18°C. Data are presented as mean ± SEM. *P*-values were calculated using two-tailed unpaired Student's *t*-test (a, b, and c) and two-way repeated-measures ANOVA with Tukey's multiple comparison post hoc test (b) following Shapiro–Wilk normality test.

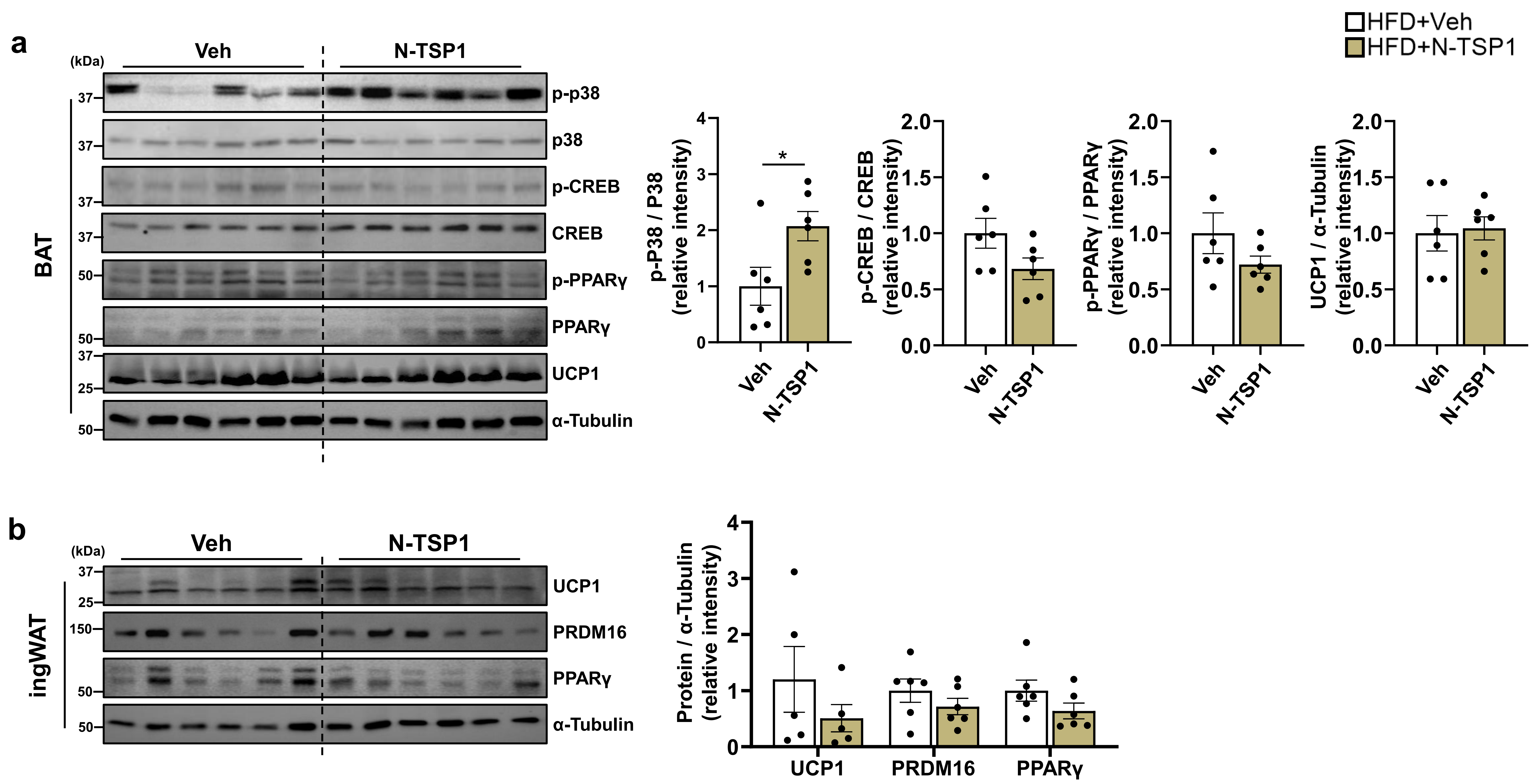


**Supplementary Fig. 6. Effects of N-TSP1 on thermogenic signaling and adipose remodeling-related markers in BAT and ingWAT of HFD-fed mice.**

Western blot analysis was performed after 8 weeks of vehicle and N-TSP1 treatment (0.5 mg/kg/day). (a) Western blot analysis of expression and phosphorylation levels of p38, CREB, PPARγ, and UCP1 in BAT (left). Relative densitometric quantification is depicted in the bar graphs (n = 6 per group) (right). (b) Western blot analysis of UCP1, PRDM16, and PPARγ expression in ingWAT (left). Relative densitometric quantification is depicted in the bar graphs (n = 6 per group) (right). Data are presented as mean ± SEM. *P*-values were calculated using two-tailed unpaired Student's *t*-test following Shapiro–Wilk normality test (a and b). (*$P < 0.05$).

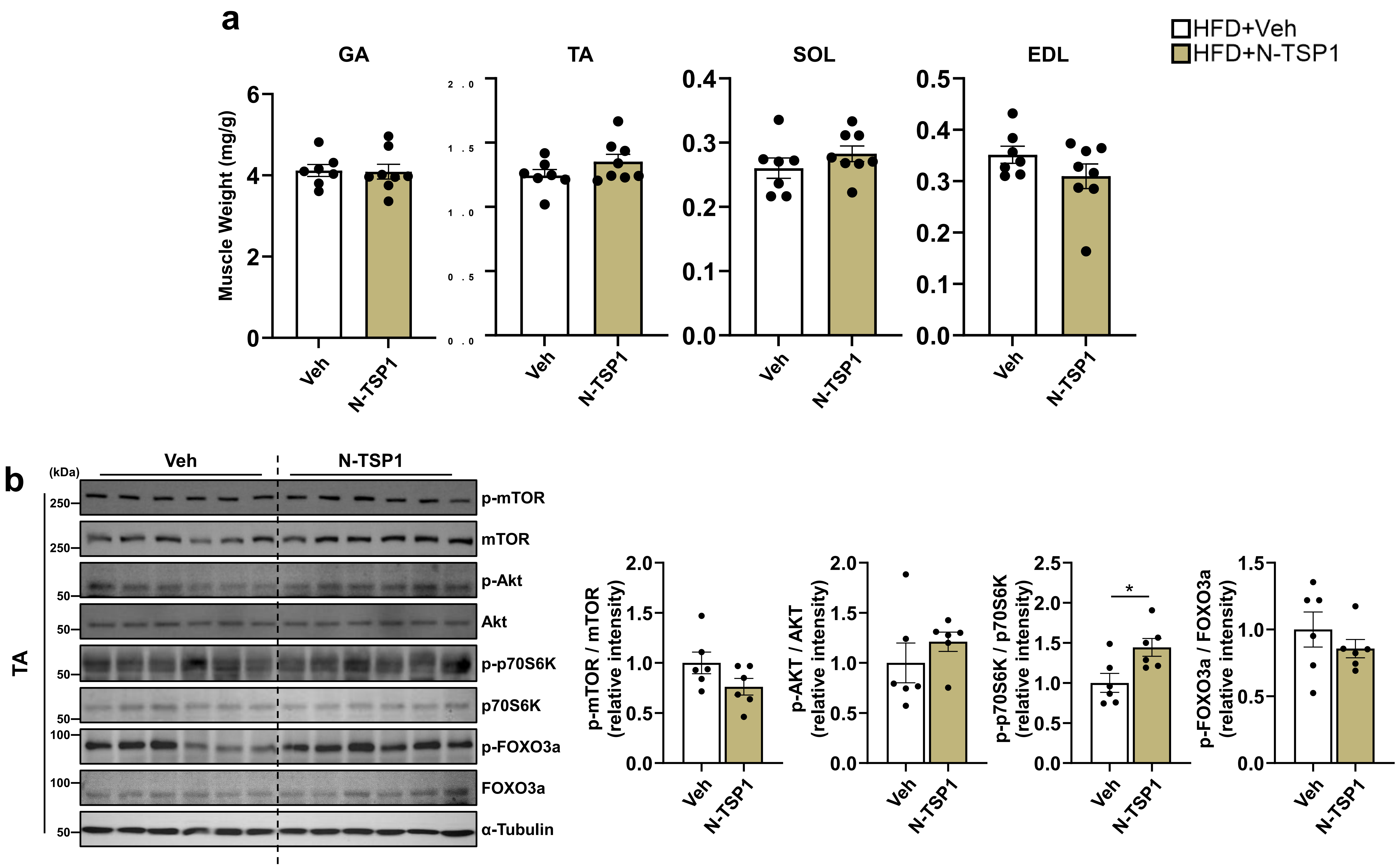


**Supplementary Fig. 7. Alterations in muscle weight and protein synthesis-related signaling pathways in mice following the administration of N-TSP1 or vehicle.**

(a) Muscle weights of HFD mice injected with vehicle or N-TSP1 (n = 8 per group). (b) Western blot analysis of expression and phosphorylation levels of proteins in the Akt/mTOR signaling pathway in tibialis anterior muscle (left). Relative densitometric quantification of panel is depicted in the bar graph (n = 6 per group) (right). Data are presented as mean ± SEM. *P*-values were calculated using two-tailed unpaired Student's *t*-test (a and b) following Shapiro–Wilk normality test. Mann–Whitney U test was employed for non-parametric data (b). (*$P < 0.05$).

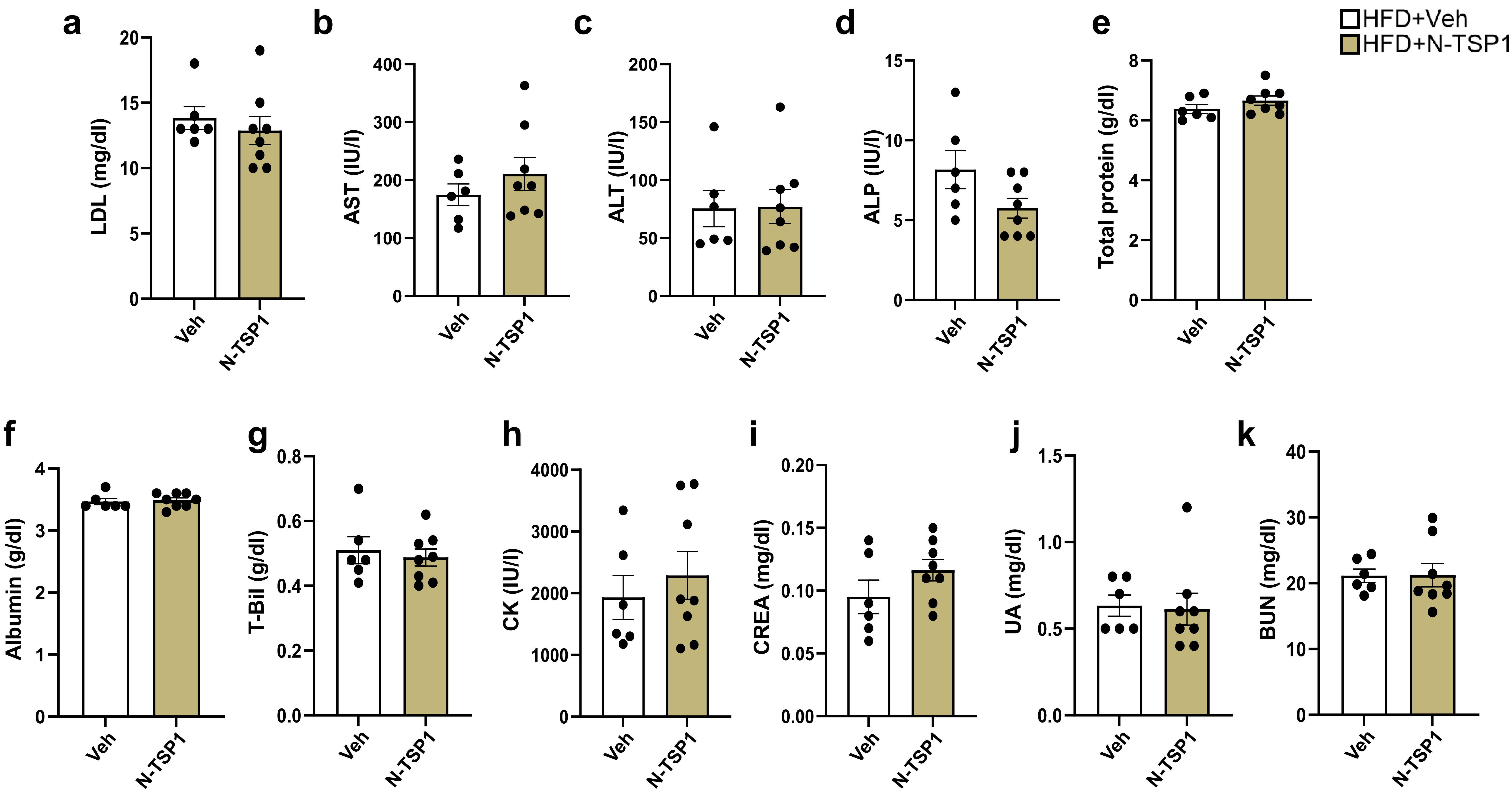


**Supplementary Fig. 8. Plasma analysis of HFD-fed mice injected with vehicle or N-TSP1.**

(a) LDL, low-density lipoprotein cholesterol; (b) AST, aspartate aminotransferase; (c) ALT, alanine aminotransferase; (d) ALP, alkaline phosphatase; (e) total protein; (f) albumin; (g) T-Bil, Total bilirubin; (h) CK, Creatine phosphokinase; (i) CREA, Creatinine; (j) UA, Uric acid; and (k) BUN, Blood urea nitrogen (n = 6–8 per group). Data are presented as mean ± SEM. *P*-values were calculated using two-tailed unpaired Student's *t*-test (b, c, d, e, g, h, i, and k) following Shapiro–Wilk normality test. Mann–Whitney U test was employed for non-parametric data (a, f, and j).

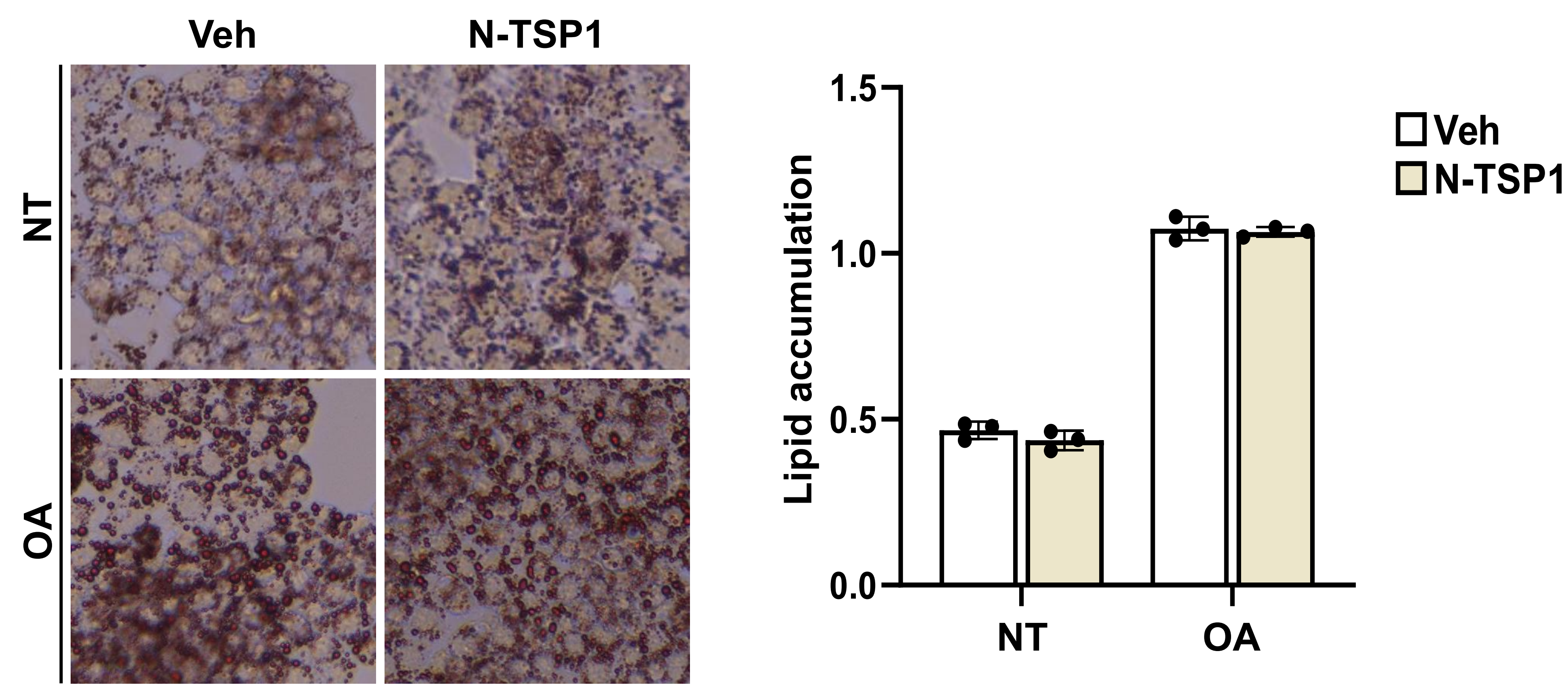


**Supplementary Fig. 9. N-TSP1 treatment does not affect lipid accumulation in HepG2 cells.**

Representative images of Oil Red O-stained HepG2 cells showing oleic acid-induced lipid accumulation in the presence or absence of N-TSP1 (left). Quantification of lipid droplets in panel (n = 3 per group) (right). Data are presented as mean ± SEM. *P*-values were calculated using two-tailed unpaired Student's *t*-test following Shapiro–Wilk normality test.

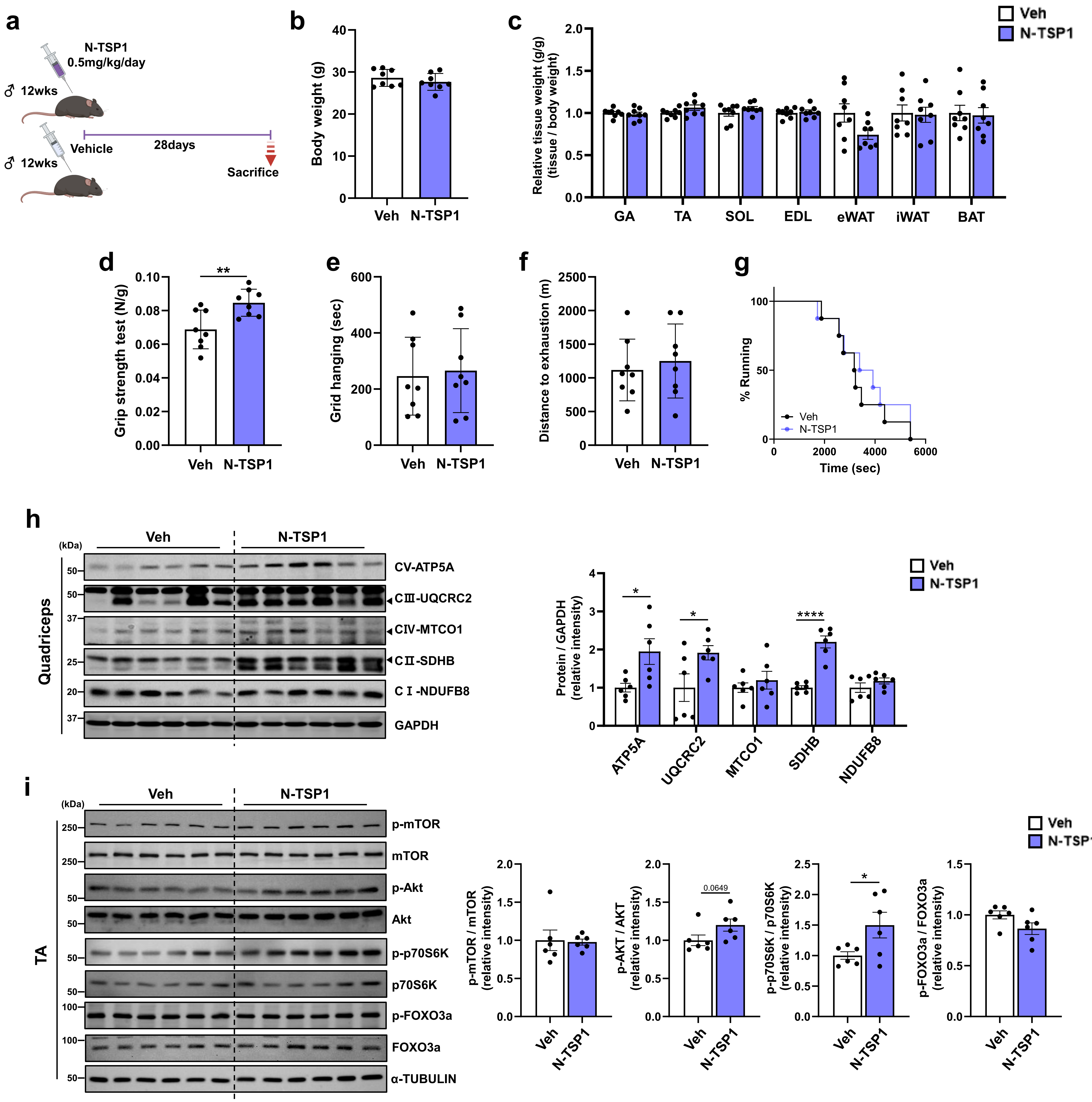
a
N-TSP1
0.5mg/kg/day
♂ 12wks
Vehicle
28days
♂ 12wks
Sacrifice
b
Body weight (g)
Veh
N-TSP1
c
Relative tissue weight (g/g)
(tissue / body weight)
GA
TA
SOL
EDL
eWAT
iWAT
BAT
Veh
N-TSP1
d
Grip strength test (N/g)
e
Grid hanging (sec)
f
Distance to exhaustion (m)
g
% Running
Time (sec)
h
Quadriceps
(kDa)
CV-ATP5A
CIII-UQCRC2
CIV-MTCO1
CII-SDHB
C I -NDUFB8
GAPDH
Protein / GAPDH
(relative intensity)
ATP5A
UQCRC2
MTCO1
SDHB
NDUFB8
i
TA
p-mTOR
mTOR
p-Akt
Akt
p-p70S6K
p70S6K
p-FOXO3a
FOXO3a
α-TUBULIN
p-mTOR / mTOR
(relative intensity)
p-AKT / AKT
(relative intensity)
0.0649
p-p70S6K / p70S6K
(relative intensity)
p-FOXO3a / FOXO3a
(relative intensity)

**Supplementary Fig. 10. Effects of N-TSP1 administration on muscle performance, protein anabolic signaling, and mitochondrial function in mice fed a standard diet.**

(a) Schematic showing N-TSP1 supplementation. Young (12 weeks old) mice were injected intraperitoneally with vehicle or N-TSP1 (0.5 mg/kg/day) for 28 days (n = 6 per group). (b–g) Physical parameters (n = 8 per group) (b) Body weight measurement. (c) Weights of skeletal muscles; GA, TA, Sol, and EDL and adipose tissues; epididymal, inguinal, and brown adipose tissues (BAT). (d) Grip strength measurement. (e) Grid-hanging time measurement. (f) Distance to exhaustion during treadmill exercise. (g) Time to exhaustion during treadmill exercise. (h) Immunoblot of OXPHOS complexes in quadriceps muscle (left). Relative densitometric quantification is depicted in the bar graph (n = 6 per group) (right). (i) Western blot analysis of expression and phosphorylation levels of AKT/mTOR signaling pathway in tibialis anterior muscle (left). Relative densitometric quantification is depicted in the bar graph (n = 6 per group) (right). Data are presented as mean ± SEM. *P*-values were calculated using two-tailed unpaired Student's *t*-test following Shapiro–Wilk normality test (b, c, d, e, f, h, and i). Mann–Whitney U test was employed for non-parametric data (c and i). Log-rank (Mantel-Cox) test was employed to calculate the *P*-value in panel g ($*P < 0.05$, $**P < 0.01$, $****P < 0.0001$).

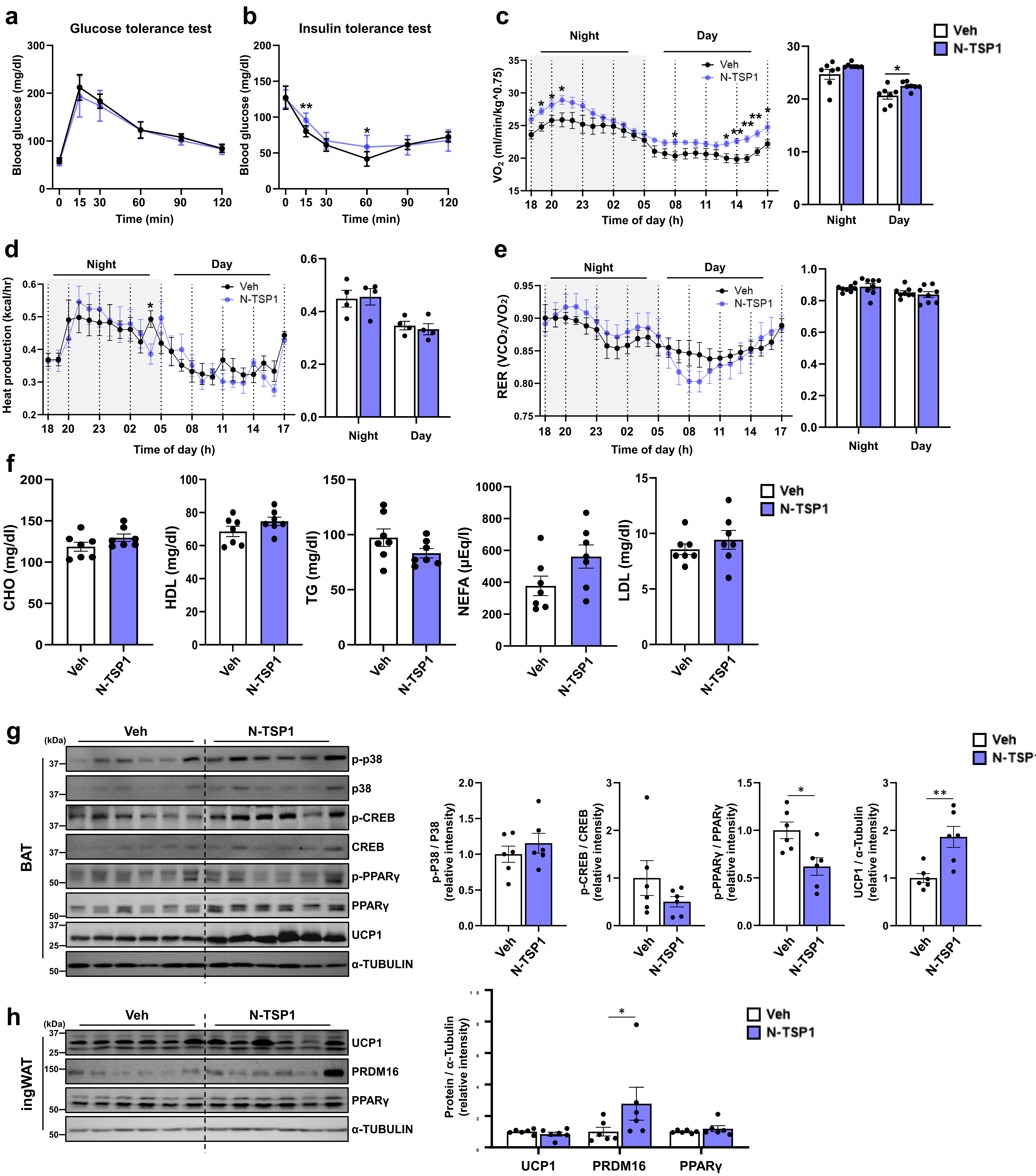
a
Glucose tolerance test
Blood glucose (mg/dl)
Time (min)
b
Insulin tolerance test
Blood glucose (mg/dl)
Time (min)
c
Night
Day
Veh
N-TSP1
VO2 (ml/min/kg^0.75)
Time of day (h)
d
Heat production (kcal/hr)
Time of day (h)
e
RER (VCO2/VO2)
Time of day (h)
f
CHO (mg/dl)
HDL (mg/dl)
TG (mg/dl)
NEFA (µEq/l)
LDL (mg/dl)
g
(kDa)
BAT
p-p38
p38
p-CREB
CREB
p-PPARγ
PPARγ
UCP1
α-TUBULIN
p-P38 / P38 (relative intensity)
p-CREB / CREB (relative intensity)
p-PPARγ / PPARγ (relative intensity)
UCP1 / α-Tubulin (relative intensity)
h
ingWAT
UCP1
PRDM16
PPARγ
α-TUBULIN
Protein / α-Tubulin (relative intensity)

**Supplementary Fig. 11. Metabolic parameters in normal chow-fed mice following N-TSP1 administration.**

(a) GTT after 12-h fasting. (b) ITT after 6 h fasting (n = 8 per group). (c) Oxygen consumption rate over 24 h (left). Average night- and day-time OCR (right). (d) Heat production during 24 h (left). Average night- and day-time heat production (right). (e) RER during 24 h (left). Average night- and day-time RER (n = 4 per group) (right). (f) Serum analysis; CHO: Total cholesterol, HDL: High-density lipoprotein, TG: Triglyceride, NEFA: Non-esterified fatty acid, LDL: Low-density lipoprotein (n = 8 per group). (g) Western blot analysis of expression and phosphorylation levels of p38, CREB, PPARγ, and UCP1 in BAT. α-Tubulin was used as a loading control (left). Relative densitometric quantification is depicted in the bar graph (n = 6 per group) (right). (h) Western blot analysis of UCP1, PRDM16, and PPARγ expression in inguinal white adipose tissue (left). Relative densitometric quantification of panel is depicted in the bar graph (n = 6 per group) (right). Data are presented as mean ± SEM. *P*-values were calculated using two-way repeated-measures ANOVA with Tukey's multiple comparison post hoc test (a, b, c, d, and e) and two-tailed unpaired Student's *t*-test (c, d, e, f, g, and h) following Shapiro–Wilk normality test. Mann–Whitney U test was employed for non-parametric data (d and h) (*$P < 0.05$, **$P < 0.01$).

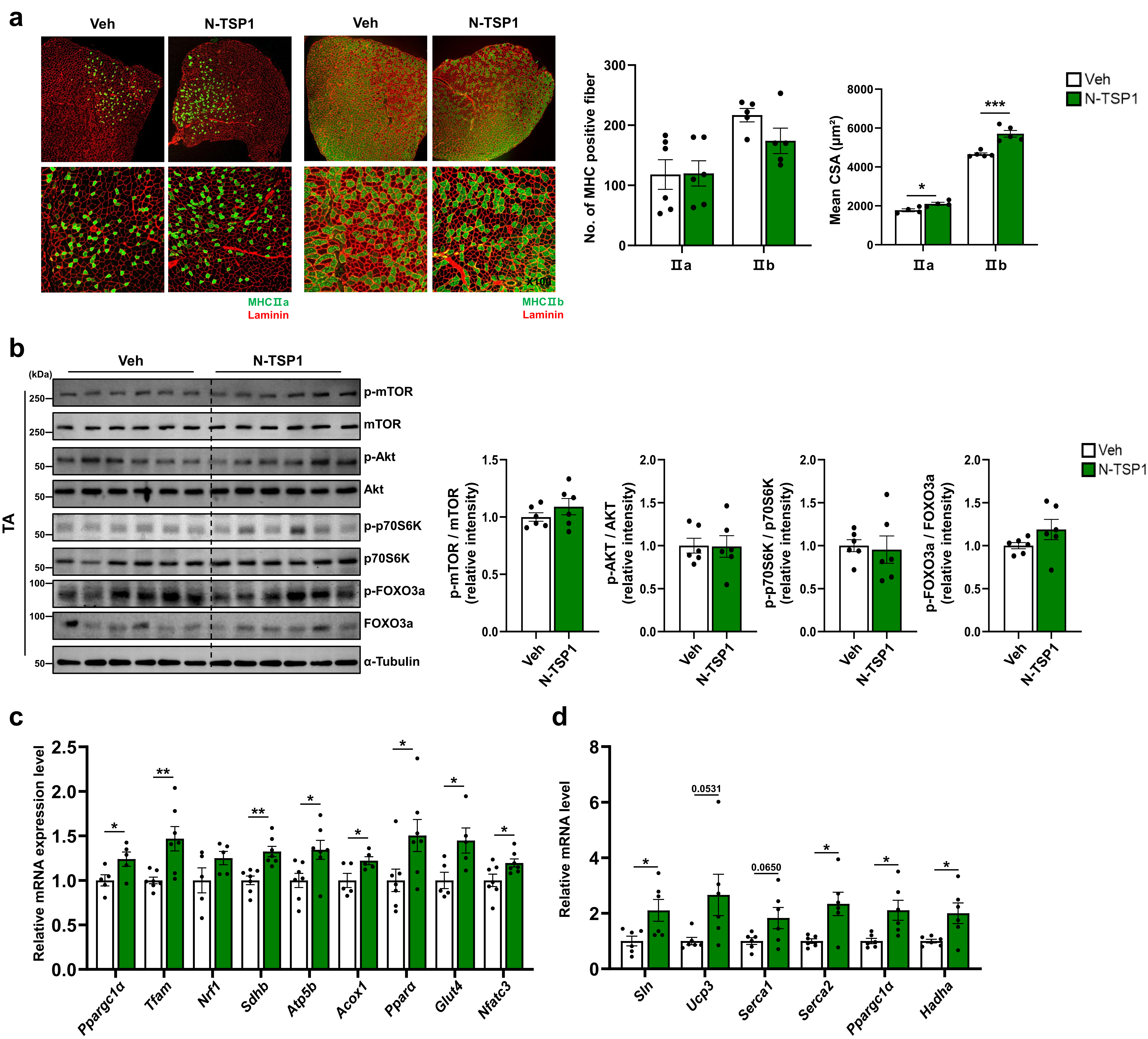

a
Veh
N-TSP1
Veh
N-TSP1
MHCⅡa
Laminin
MHCⅡb
Laminin
x100
No. of MHC positive fiber
Mean CSA (μm²)
Ⅱa
Ⅱb
Veh
N-TSP1
b
Veh
N-TSP1
(kDa)
TA
p-mTOR
mTOR
p-Akt
Akt
p-p70S6K
p70S6K
p-FOXO3a
FOXO3a
α-Tubulin
p-mTOR / mTOR (relative intensity)
p-AKT / AKT (relative intensity)
p-p70S6K / p70S6K (relative intensity)
p-FOXO3a / FOXO3a (relative intensity)
c
Relative mRNA expression level
Ppargc1α
Tfam
Nrf1
Sdhb
Atp5b
Acox1
Pparα
Glut4
Nfatc3
d
Relative mRNA level
0.0531
0.0650
Sln
Ucp3
Serca1
Serca2
Ppargc1α
Hadha

**Supplementary Fig. 12. N-TSP1 promotes muscle fiber remodeling and enhances thermogenic and metabolic adaptation in aged skeletal muscle.**

(a) Immunostaining of MyHCIIa (green) (left), MyHCIIb (green) (right), and laminin (red) in the TA muscles from 23-month-old mice injected with vehicle or N-TSP1 (0.5 mg/kg for 15 days) (left). Quantification of MyHCIIa- and MyHCIIb-positive myofibers and cross-sectional area (CSA) (n = 3 per group) (right). (b) Immunoblots of expression and phosphorylation levels of proteins in the AKT/mTOR signaling pathway in tibialis anterior muscle of aged mice (left). Relative densitometric quantification is depicted in the bar graph (right) (n = 6 per group). (c) Relative mRNA expression of mitochondrial function-related genes in the quadriceps muscle (n = 6 per group). (d) Relative mRNA expression of thermogenic and mitochondrial metabolic regulation-related genes (n = 6 per group). Data are presented as mean ± SEM. *P*-values were calculated using two-tailed unpaired Student's *t*-test (a, b, c, and d) following Shapiro–Wilk normality test (*$P < 0.05$, ** $P < 0.01$, ***$P < 0.001$).

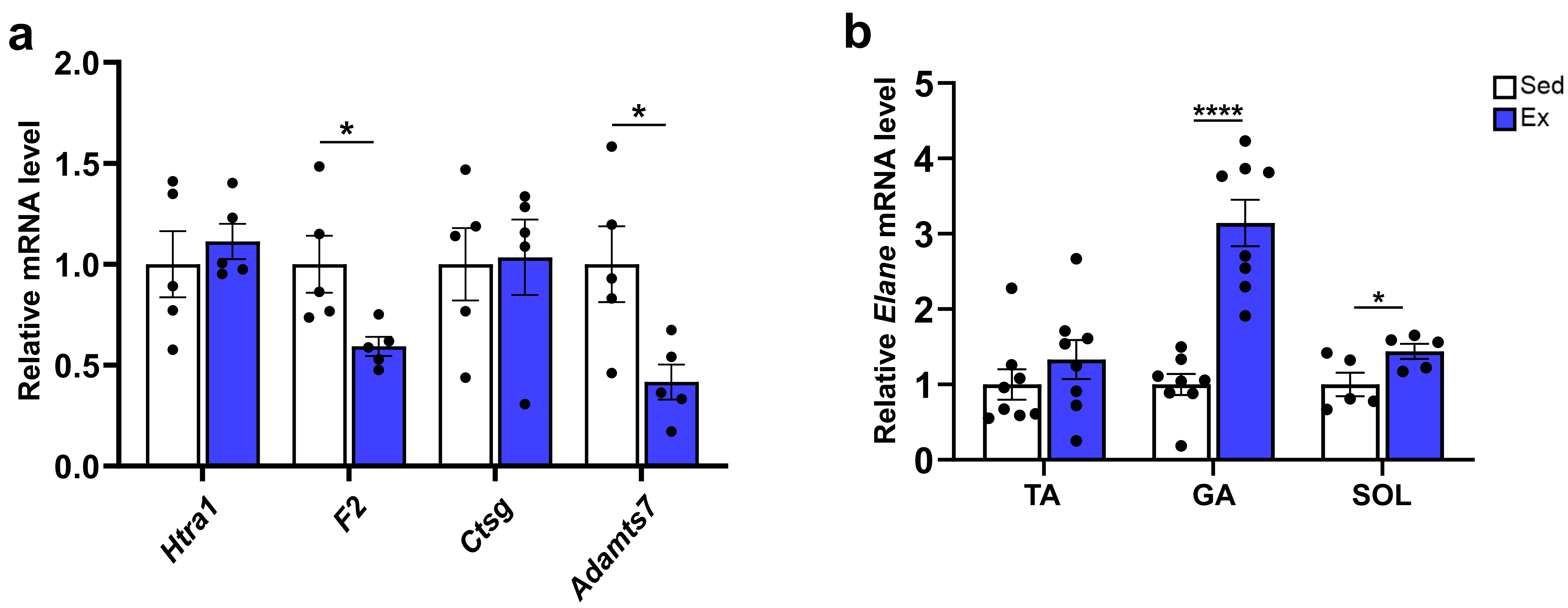


**Supplementary Fig. 13. Effects of exercise on the expression of proteases implicated in TSP1 cleavage and N-TSP1 generation.**

(a) Relative mRNA expression of proteases known to cleave TSP1 in the soleus muscle of treadmill-exercised mice. The analyzed proteases include HtrA1 (encoded by *Htra1*), thrombin (encoded by *F2*), cathepsin G (encoded by *Ctsg*), ADAMTS7 (encoded by *Adamts7*) (n = 5 per group). (b) Relative mRNA expression of *Elane*, encoding neutrophil elastase (ELANE) in the TA, GA, and SOL muscles of treadmill-exercised mice (n = 6–8 per group). Data are presented as mean ± SEM. *P*-values were calculated using two-tailed unpaired Student's *t*-test following Shapiro–Wilk normality test. Mann–Whitney U test was employed for non-parametric data (a and b). (*$P < 0.05$, ****$P < 0.0001$).

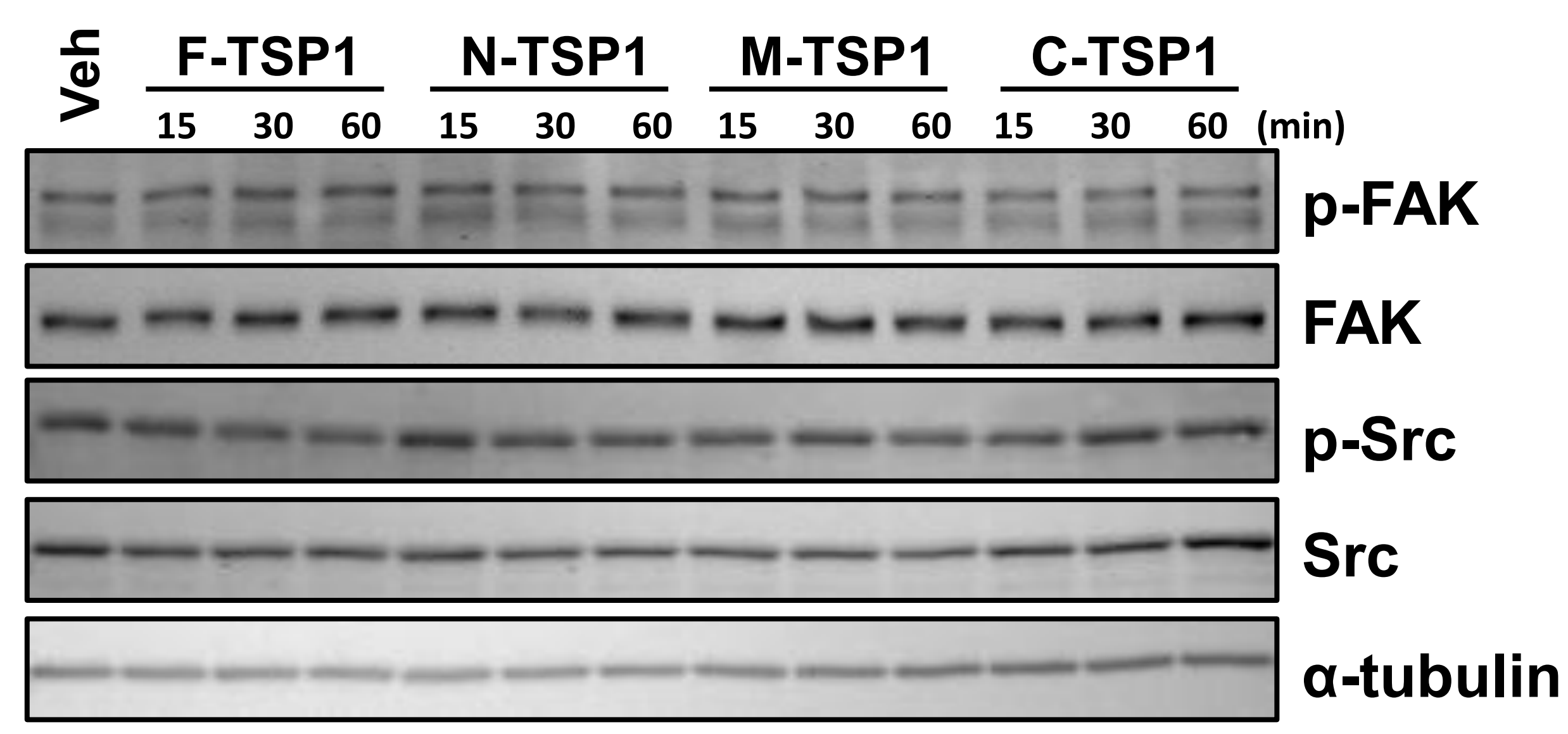


**Supplementary Fig. 14. N-TSP1 does not activate integrin signaling in C2C12 myotubes.**

Western blot analysis of expression and phosphorylation levels of FAK and Src. C2C12 myotubes were treated with F-TSP1, N-TSP1, M-TSP1, or C-TSP1 (1μg/mL) after 6 h of serum starvation.
